# Unconventional CD8^+^ T cell surveillance of cytomegalovirus via Qa-1/HLA-E–restricted epitope recognition

**DOI:** 10.1126/sciadv.aea8707

**Published:** 2025-12-19

**Authors:** Shanelle P. Reilly, Madison L. Smith, Samantha M. Borys, Céline Fugère, Delia Demers, Michael J. Hogan, David Zemmour, Laurent Brossay

**Affiliations:** ^1^ Department of Molecular Microbiology and Immunology, Division of Biology and Medicine, Brown University, Providence, RI 02906, USA.; ^2^ Department of Pathobiology, School of Veterinary Medicine, University of Pennsylvania, Philadelphia, PA 19104, USA.; ^3^ Department of Pathology, University of Chicago, Chicago, IL 60637, USA.

## Abstract

Nonclassical CD8^+^ T cells can compensate for classical CD8^+^ T cell effector responses during murine cytomegalovirus (MCMV) infection. Through a combination of motif-based discovery, predictive algorithms, AlphaFold3 structural modeling, and biological assays, we identified multiple MCMV and human cytomegalovirus (HCMV) peptides that bind to Qa-1 and HLA-E, respectively. In the mouse system, we demonstrated that these virally encoded antigens stimulate Qa-1–restricted CD8^+^ T cells ex vivo, which can be tracked using MCMV peptide–loaded Qa-1 tetramers. Adoptive transfer of predominantly Qa-1 tetramer^+^ CD8^+^ T cells into RAG-1–deficient mice protects them from mortality, underscoring the critical role of these cells in host defense. Single-cell RNA (scRNA)/TotalSeq and single-cell T cell receptor sequencing (scTCR-seq) reveal the expansion of unique TCR αβ clonotypes, indicating convergent antigen specificity. Together, our findings uncover a conserved and functionally important nonclassical CD8^+^ T cell axis mediated by Qa-1/HLA-E modulating adaptive immunity independent of classical major histocompatibility complex class I (MHC-I) pathways and present previously unidentified opportunities for vaccine development.

## Teaser

A recently identified group of CD8^+^ T cells helps fight cytomegalovirus using a conserved immune recognition pathway.

## INTRODUCTION

Human cytomegalovirus (HCMV) is a ubiquitous double-stranded DNA (dsDNA) β-herpesvirus that establishes latency and develops a long-term evolutionary relationship with the host (*1*). Seroprevalence exceeds 80% globally and correlates with age, socioeconomic status, and education level (*2*, *3*). In immunocompetent individuals, primary infection is typically asymptomatic and controlled efficiently, followed by lifelong latency and intermittent subclinical reactivation (*4*). Immunity against CMV is multifaceted, requiring contributions from both innate and adaptive arms. Host immune responses are initially broad, and a wide breadth of antigen-specific responses, rather than the magnitude of the T cell response, appears to be more effective at keeping viral titers low (*5*). In particular, memory T cell responses are crucial for maintaining latency and controlling viral reactivation (*6*). However, HCMV remains a major cause of morbidity and mortality in immunocompromised populations, including congenitally infected neonates and transplant recipients (*7*–*9*). The development of an effective CMV vaccine remains a critical unmet need, with the potential to prevent congenital disease and reduce complications in high-risk patients.

A large portion of the CMV genome is dedicated to host immune evasion. The viral genome encodes numerous proteins that modulate host major histocompatibility complex class I (MHC-I) pathways (*10*, *11*), allowing the virus to escape CD8^+^ T cell detection while concurrently limiting natural killer (NK) cell–mediated cytotoxicity through the expression of MHC-I homologs (*12*, *13*). Murine cytomegalovirus (MCMV) serves as a valuable model for dissecting CMV-host dynamics, mirroring many aspects of HCMV, including viral latency, immune evasion, and epitope-specific T cell responses (*14*). Among classical CD8^+^ T cell responses to MCMV, the viral epitope M45 presented by H2-D^b^ is well characterized and immunodominant (*14*). In contrast, nonclassical CD8^+^ T cell responses restricted by MHC-Ib molecules, such as Qa-1 in mice and HLA-E in humans, remain less defined. Despite increasing interest in nonclassical CD8^+^ T cells, the molecular mechanisms underlying their activation, antigen specificity, and contribution to long-term immune protection remain incompletely defined. Elucidating these pathways may reveal previously unidentified strategies to bolster antiviral immunity, especially in contexts where classical MHC-I–restricted responses are subverted or dysfunctional.

HLA-E, one of the least polymorphic MHC-I molecules, is predominantly expressed as two dominant alleles (HLA-E*01:01 and HLA-E*01:03), differing by one amino acid at position 107 (Arg^107^ → Gly) (*15*). Under homeostatic conditions, Qa-1 and HLA-E present conserved signal peptides from classical MHC-I molecules: Qdm (AMAPRTLLL) and VL9 (VMAPRTLIL/LLL/VLL), respectively (*16*–*18*). Endoplasmic reticulum aminopeptidase (ERAAP)–deficient cells injected in wild-type (WT) mice elicit a robust cytotoxic CD8^+^ T cell response, due to presentation of the self-peptide FL9 (FYAEATPML) by Qa-1 (*19*–*21*). Emerging evidence indicates that Qa-1 and HLA-E can present microbial and viral peptides to cytotoxic CD8^+^ T cells. Qa-1–restricted T cell responses have also been documented in infections with *Listeria monocytogenes*, *Salmonella typhimurium*, and *Mycobacterium tuberculosis* (*22*, *23*), as well as during influenza infection (*24*) and MCMV infection (*25*, *26*).

Here, we identify and functionally characterize Qa-1–binding MCMV peptides that activate nonclassical CD8^+^ T cells. Using predictive modeling, tetramer staining, and ex vivo functional assays, we show that these Qa-1–restricted responses emerge during both acute and chronic phases of infection, are antigen-dependent, and persist in tissue sites of viral latency, particularly the immune-privileged salivary gland. We further demonstrate that submandibular gland (SMG)–resident, Qa-1–restricted CD8^+^ T cells confer protection in immunodeficient hosts, representing the only model in which MHC-E–restricted CD8^+^ T cells have been shown to mediate pathogen protection. By integrating single-cell transcriptomics, cellular indexing of transcriptomes and epitopes by sequencing (CITE-seq), and T cell receptor (TCR) repertoire analysis, we reveal a clonally expanded, antigen-experienced population with an exhaustion-like phenotype. Last, extending our findings to humans, we identify multiple HCMV-encoded peptides capable of binding HLA-E, highlighting a conserved, functionally relevant axis of antiviral immunity mediated by nonclassical MHC molecules.

## RESULTS

### Several MCMV peptides bind to Qa-1 and share anchor residues with known Qa-1 binders

**Fig. 1.**
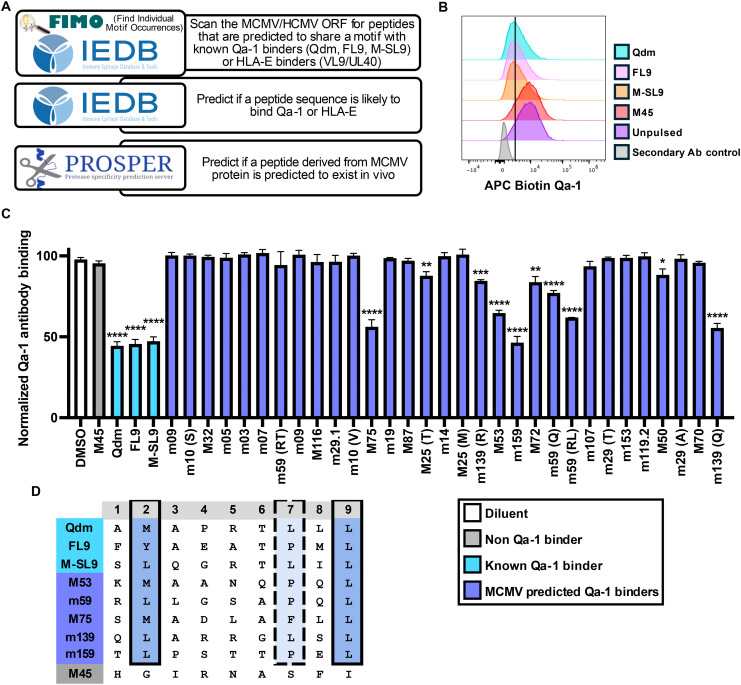
Several MCMV peptides bind to Qa-1 and share anchor residues with known Qa-1 binders. (**A**) Schematic of the bioinformatics workflow used to generate the MCMV peptide library listed in table S1. (**B**) P815 cells were pulsed with peptides (non–Qa-1 binder M45 and known binders Qdm, FL9, and M-SL9), and Qa-1 staining was performed. Histograms are representative of eight independent experiments (gating strategy available in fig. S1). (**C**) Normalized Qa-1 antibody binding on the surface of P815 cells following incubation with each indicated peptide or dimethyl sulfoxide (DMSO) control. Peptides derived from the same MCMV protein are denoted by the first letter(s) of the peptide sequence in parentheses following the viral protein name. Data are pooled from eight independent experiments, *n* = 3 to 8 per group. Data are presented as the means ± standard error of the mean (SEM). Statistical significance was determined using a one-way analysis of variance (ANOVA), and significant differences are *P < 0.05; **P < 0.01; ***P < 0.001; ****P < 0.0001. (**D**) Peptide sequence schematic highlighting anchor positions between known Qa-1 binders and MCMV peptides. Solid lines indicate the same or extremely similar amino acid (AA) usage, while dotted lines indicate somewhat similar AA usage, based on biochemical properties. The strongest m59 peptide, m59(RL), is indicated as the two m59 peptides differed by one AA.

HCMV has one of the largest known genomes of any virus to infect humans, roughly 236 kilo–base pairs (kbp) (*27*). The genome of MCMV is colinear to that of HCMV and is roughly 230 kbp (*28*). MCMV and HCMV have been shown to encode functionally analogous immune evasion genes (*29*). A recent study illustrated the expansion of Qa-1-FL9–restricted (QFL) CD8^+^ T cells during the early phase of MCMV infection, following viral down-regulation of ERAAP (*26*). We reasoned that antigen(s) presented by Qa-1 during infection share a motif with Qdm or FL9 and used both peptide sequences as input for FIMO (Find Individual Motif Occurrences), a software tool that uses input sequences to scan protein sequences for similar motifs (Fig. 1A) (*30*). Other peptide sequences known to bind to Qa-1 were also examined, including the flu-derived cryptic epitope M-SL9 (*24*). The leader sequences Qdm and VL9 can bind to both Qa-1 and HLA-E with strong affinity (*31*). Both MHC molecules display a preference for binding epitopes that maintain hydrophobic residues at P2, P9, and often P7 (*31*, *32*). Therefore, we also included VL9, which shares 100% sequence identity with HCMV-encoded UL40, in motif discovery. The Immune Epitope Database Analysis Resource (IEDB-AR) contains T cell epitope prediction algorithms that have been trained on validated peptide-MHC binding data (*33*). In parallel to FIMO analysis, we also used NetMHCpan EL (epitope prediction) to identify candidate peptide sequences from the MCMV genome that might be naturally processed and presented by Qa-1 (*34*). Top hits found both via this method and FIMO were selected (Fig. 1A). We then tested whether peptide sequences were predicted to bind Qa-1 and HLA-E using the MHC-I Binding Prediction software, also available from IEDB-AR. NetMHCpan BA (binding prediction) incorporated the likelihood of each peptide to bind Qa-1(b), HLA-E*01:01, or HLA-E*01:03, respectively (*34*). Priority was given to peptides with binding scores highest for Qa-1 first, then HLA-E (table S1). Last, to eliminate peptides predicted to be destroyed by a cleavage site within MCMV proteins, we used the in silico approach PROSPER (protease specificity prediction server) (*35*). A final peptide library was generated from these collective bioinformatic approaches (table S1).

Once the MCMV peptide library was generated, we tested whether these peptides were able to bind to Qa-1. To do this, we used the mast cell line P815, which we found expresses high levels of Qa-1 on its cell surface (fig. S1). The anti–Qa-1 monoclonal antibody (mAb) used in this study has been reported to have decreased binding when Qa-1 is loaded with peptides (*36*). Therefore, peptide binders lead to decreased antibody binding. Although the M45 peptide is presented by the MHC-I molecule H2-D^b^, it did not bind to Qa-1 expressed on P815 (Fig. 1, B and C). Conversely, when incubated with known Qa-1 binders, including Qdm, FL9, and M-SL9, Qa-1 antibody binding decreased by about 50% (Fig. 1, B and C). We next tested our MCMV peptide library and found several MCMV peptides bound Qa-1 as strongly as canonical binders, including peptides derived from M53, m59, M75, m139, and m159 proteins (Fig. 1C). Expectedly, these peptide sequences maintained large hydrophobic residues at P2 (Met or Leu) and P9 (Leu) and displayed hydrophobic as well as rigid residues (Pro) at P7 (Fig. 1D). When modeled using AlphaFold3 (*37*), these Qa-1 binders all displayed a predicted local distance difference test (pLDDT) confidence score of over 80, indicating a high level of confidence in the accuracy of the predicted structure of each peptide within the peptide binding groove (fig. S1D).

### MCMV peptides elicit cytokine production from Qa-1–restricted CD8^+^ T cells

**Fig. 2.**
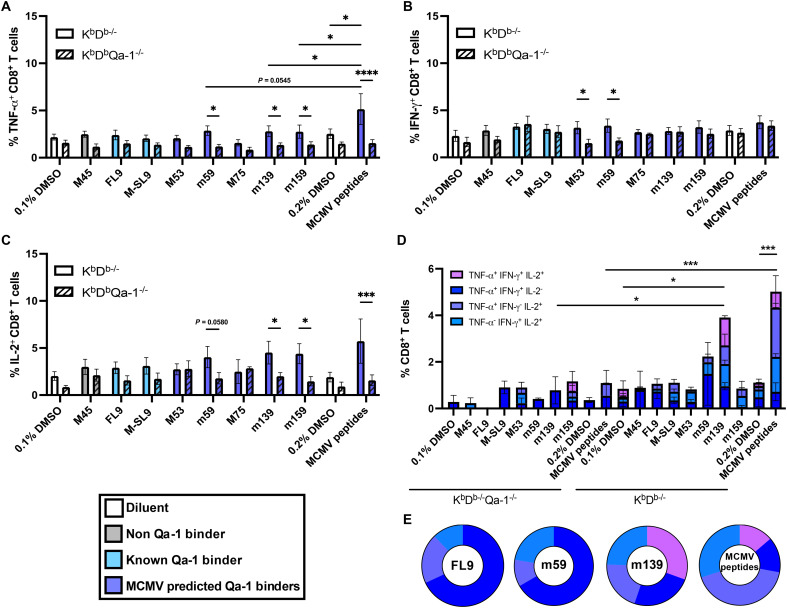
MCMV peptides elicit cytokine production in Qa-1–restricted CD8^+^ T cells. Long-term infected K^b^D^b−/−^ and K^b^D^b−/−^Qa-1^−/−^ mice were reinfected with 5 × 10^4^ PFU MCMV-RVG102. Splenocytes were stimulated with the indicated peptide(s) and incubated for 6 hours in the presence of monensin. (**A**) TNF-α, (**B**) IFN-γ, or (**C**) IL-2 cytokine responses in CD8^+^ T cell populations were measured. (**D**) Stacked representation of populations eliciting two or three cytokines. (**E**) Pie charts represent mean proportions of populations that secrete at least two cytokines. Data in (A) is pooled from four independent experiments, *n* = 8 to 9 per group. Data in [(B) and (C)] are pooled from three independent experiments, *n* = 6 to 7 per group. Data in [(D) and (E)] are representative of two independent experiments. Data are presented as the means ± SEM. Statistical analysis for [(A) to (D)] used a two-way ANOVA with Tukey’s multiple comparisons test. Significant differences are *P < 0.05; ***P < 0.001; ****P < 0.0001.

To assess whether Qa-1 loaded with MCMV peptides can induce a CD8^+^ T cell response, we developed an ex vivo stimulation assay using infected K^b^D^b−/−^ and K^b^D^b−/−^Qa-1^−/−^ mice. We found that CD8^+^ T cells from infected K^b^D^b−/−^ mice but not K^b^D^b−/−^Qa-1^−/−^ mice secrete several inflammatory cytokines following incubation with MCMV peptides (Fig. 2, A to C). Specifically, peptides derived from m59, m139, and m159 triggered tumor necrosis factor–α (TNF-α) in CD8^+^ T cells from K^b^D^b−/−^ but not from K^b^D^b−/−^Qa-1^−/−^ mice (Fig. 2A), demonstrating that these viral peptides were presented by Qa-1. The production of interferon-γ (IFN-γ) by Qa-1–restricted CD8^+^ T cells was limited, with only M53 and m59 peptides eliciting a response above background (Fig. 2B), whereas m139, m159, and, to a lesser extent, m59 triggered interleukin-2 (IL-2) production (Fig. 2C). Stimulation by all MCMV peptides concurrently resulted in an additive effect in both TNF-α– and IL-2–producing T cells (Fig. 2, A and C). Remarkably, m139-specific CD8^+^ T cells displayed a high level of cytokine polyfunctionality, including a subset expressing all three cytokines, which was also observed in Qa-1–restricted CD8^+^ T cells stimulated by all MCMV peptides (Fig. 2, D and E). Together, these data demonstrate that the CD8^+^ T cell response to MCMV peptides m59, m139, and m159 is mediated by Qa-1. Notably, the self-peptide FL9 and the flu peptide M-SL9, which bind to Qa-1, did not induce a cytokine response above background following reinfection in K^b^D^b−/−^ mice (Fig. 2, A to D).

### m59- and m139-specific CD8^+^ T cells contribute to the viral immune response

**Fig. 3.**
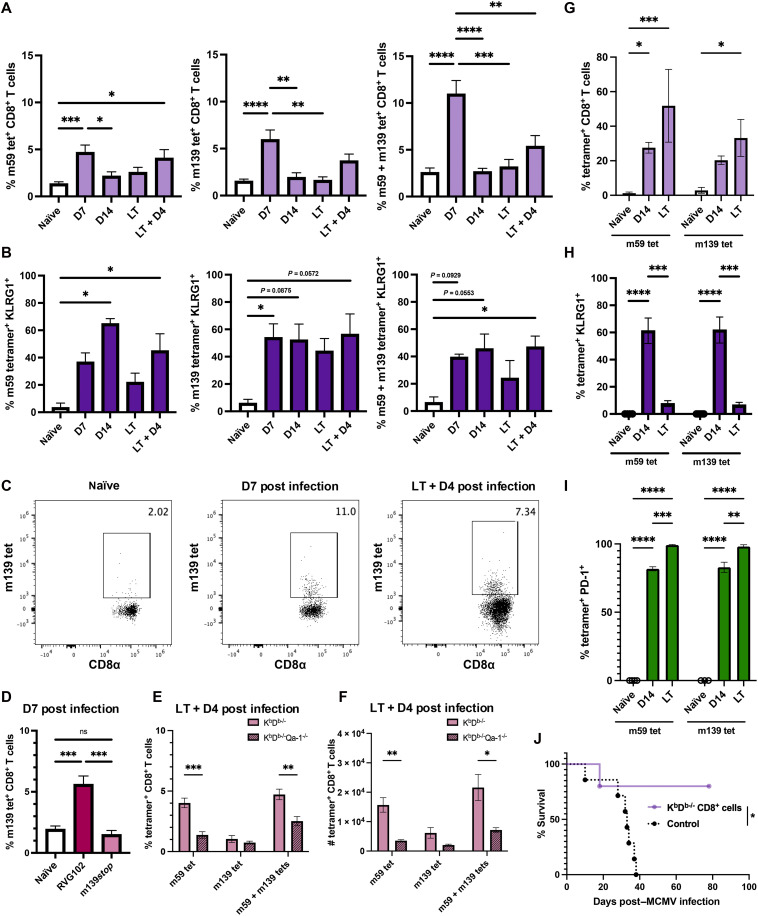
m59- and m139-specific CD8^+^ T cells contribute to the viral immune response and protect against MCMV-induced lethality. (**A**) Tetramer (tet)^+^ CD8^+^ T cell frequency in spleens from naïve and infected K^b^D^b−/−^ mice. Data are pooled from 8 to 10 independent experiments, *n* = 3 to 13. (**B**) KLRG1^+^ tetramer^+^ CD8^+^ T cell frequency. Data are pooled from six to seven independent experiments, *n* = 3 to 5. (**C**) Representative tetramer staining of infected K^b^D^b−/−^ mice. (**D**) Splenic Qa-1-m139 tetramer^+^ CD8^+^ T cell frequency following infection. Data are pooled from two independent experiments, *n* = 4 to 6. (**E**) Frequencies and (**F**) numbers of splenic Qa-1-m59, Qa-1-m139, and combined tetramer^+^ cells of m139*stop* infected mice. Data are pooled from three independent experiments, *n* = 6 to 9. (**G**) Qa-1-m59 and Qa-1-m139 tetramer^+^ CD8^+^ T cell frequency in SMGs from naïve or infected K^b^D^b−/−^ mice. (**H**) KLRG1 and (**I**) PD-1 frequency within SMG Qa-1-m59 or Qa-1-m139 tetramer^+^ CD8^+^ T cells. Data are representative of five independent experiments, *n* = 3 to 6. (**J**) Survival of K^b^D^b−/−^RAG-1^−/−^ mice with (—) or without (––) transfer of 5 × 10^4^ or 1.5 × 10^4^ SMG CD8^+^ T cells from long-term infected K^b^D^b−/−^ mice (*n* = 6). Data are pooled from two independent experiments, *n* = 5 to 7. Data are presented as the means ± SEM. Statistical analysis for [(A) to (C)] used a one-way ANOVA, and significant differences are *P < 0.05; **P < 0.01; ***P < 0.001; ****P < 0.0001. Statistical analysis for [(E) and (F)] used multiple unpaired *t* tests, and significant differences are **q < 0.01 or ***q < 0.001. Statistical analysis for [(G) to (I)] used a two-way ANOVA with Tukey’s multiple comparison test. Significant differences are *P < 0.05; **P < 0.01; ****P < 0.0001. Statistical analysis for (J) used a Mantel-Cox test. D7, day 7; D14, day 14; LT, long-term; LT + D4, long-term + day 4.

To track the corresponding CD8^+^ T cells in vivo, Qa-1-m59, Qa-1-m139, and Qa-1-m159 peptide-loaded tetramers were generated. Unfortunately, Qa-1-m159 peptide tetramers were unstable and could not be tested. Because Qa-1 loaded with the Qdm peptide binds to CD94/NKG2A (*38*), we performed tetramer staining in the presence and absence of an NKG2A antibody. As previously reported (*39*), we found that Qa-1-Qdm loaded tetramers bind the CD94/NKG2A receptor on NK cells (fig. S2, A and B). In contrast, we fnd that Qa-1-m59 and Qa-1-m139 tetramers did not bind to NKG2A (fig. S2B) and therefore exclusively bind to respective TCRs. We next determined the preponderance of Qa-1-m59 and Qa-1-m139 tetramer-specific CD8^+^ T cells during acute infection, long-term infection, and reinfection. We found that m59- and m139-specific CD8^+^ T cells significantly expand following acute MCMV infection (Fig. 3A). Notably, following acute infection, m59- and m139-specific CD8^+^ T cell tetramer staining displayed an additive effect, indicating that these populations are distinct from one another (Fig. 3A and fig. S2C). Furthermore, a large proportion of m59- and m139-specific CD8^+^ T cells displayed effector markers, including high expression of KLRG1 (killer cell lectin-like receptor subfamily G1) at various time points following infection (Fig. 3B). Together, these data indicate that m59- and m139-specific CD8^+^ T cells play an active role in the immune response to acute MCMV infection.

### Specificity of Qa-1-m139 CD8^+^ T cells

To validate the significance of the identified Qa-1–restricted CD8^+^ T cells, we acquired MCMV m139*stop*, a mutant MCMV that encodes for an early stop codon in the *m139* gene (*40*) and consequently lacks the m139-derived epitope. We found that while a robust population of m139-specific CD8^+^ T cells could be identified following infection of K^b^D^b−/−^ mice with WT MCMV (Fig. 3, A, C, and D), it was virtually absent following infection with MCMV m139*stop* (Fig. 3, D to F). Notably, while expansion of m139-specific CD8^+^ T cells was lost following primary infection and reinfection with MCMV m139*stop*, m59-specific CD8^+^ T cells could still be detected (Fig. 3, E and F). This demonstrates that loss of m139-specific T cells does not impair the overall CD8^+^ T cell response, including other Qa-1–restricted T cells. Both m139-specific CD8^+^ T cells and m59-specific CD8^+^ T cells were absent in K^b^D^b−/−^Qa-1^−/−^ mice infected with MCMV m139*stop* (Fig. 3, E and F). Together, these data illustrate that the viral m139 protein encodes an epitope presented to Qa-1–restricted CD8^+^ T cells during MCMV infection.

### SMG tetramer^+^ CD8^+^ T cells protect against MCMV-induced lethality

We next investigated whether Qa-1-m59 and Qa-1-m139 tetramer-specific CD8^+^ T cells were detected in the salivary gland, as it is the primary site of terminal latency for MCMV infection. MCMV traffics to the submandibular gland (SMG) between day 7 and day 14 postinfection (*41*). We found that a large proportion of SMG CD8^+^ T cells were m59- and m139-specific at both day 14 postinfection and following long-term infection in K^b^D^b−/−^ mice (Fig. 3G). At day 14 postinfection, ~60% of m59 tetramer^+^ and m139 tetramer^+^ CD8^+^ T cells expressed KLRG1, and this expression was restored to near naïve levels following long-term infection (Fig. 3H). In contrast, ~80% of m59- and m139-positive CD8^+^ T cells expressed PD-1 (programmed cell death protein 1) at day 14, and nearly all cells in these subsets were PD-1 positive at the long-term time point (Fig. 3I). These results suggest that SMG-derived m59- and m139-specific CD8^+^ T cells play an immunodominant role following MCMV infection.

To test the functionality of these subsets following terminal latency in the SMG, we used an adoptive transfer model to assess the protective capabilities of T cells in this compartment. Due to low CD8^+^ T cell populations in the SMG of K^b^D^b−/−^ mice, we pooled the SMGs from six mice per group following long-term MCMV infection. Magnetically enriched CD8^+^ T cells (95%) were transferred into simultaneously MCMV-infected K^b^D^b−/−^RAG-1^−/−^ mice. While K^b^D^b−/−^RAG-1^−/−^ control animals succumbed to infection due to the lack of adaptive immunity (Fig. 3J), adoptive transfer of SMG-derived CD8^+^ T cells resulted in 80% survival, indicating a substantial protective effect. Together, these data suggest that m59- and m139-specific CD8^+^ T cells contribute to the protective effects of SMG-derived CD8^+^ T cells in RAG-1–deficient mice.

### Unique nonclassical T cell clusters expand following MCMV infection

**Fig. 4.**
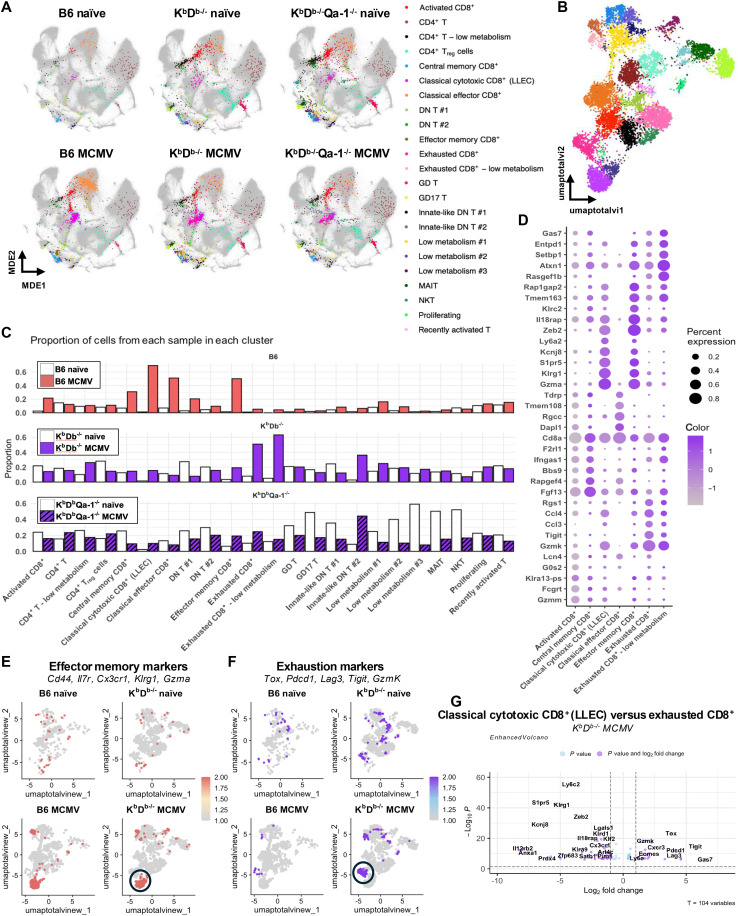
Nonclassical T cells display unique subsets following MCMV infection. B6, K^b^D^b−/−^, and K^b^D^b−/−^Qa-1^−/−^ mice were infected with MCMV. Following long-term infection, splenic CD45^+^ CD3^+^ cells were sorted from naïve and infected mice and subjected to CITE-seq/TotalSeq. (**A**) Clusters derived from these experiments projected onto a 2D-UMAP visualization of both protein and RNA expression clusters generated from totalVI integration of all 96 ImmGen T single-cell experiments. (**B**) 2D-UMAP visualization of all T cells from naïve and MCMV infected B6, K^b^D^b−/−^, and K^b^D^b−/−^Qa-1^−/−^ mice. (**C**) Bar graph representing the proportion of cells per cluster from naïve and MCMV infected WT B6, K^b^D^b−/−^, and K^b^D^b−/−^Qa-1^−/−^ mice. (**D**) Dot plot of enriched genes in each CD8^+^ cluster. The dot size represents the proportion of cells in a cluster that express a given feature, whereas the color indicates the average expression level of that feature across all cells in the cluster (with dark purple representing high expression and light purple indicating low expression). Module score of key genes associated with (**E**) effector memory functions or (**F**) exhaustion. (**G**) Volcano plot of differentially expressed genes in the classical cytotoxic CD8^+^ (LLEC) T cell cluster compared to the exhausted CD8^+^ T cell cluster in K^b^D^b−/−^ mice. Plots created using Seurat (v5). MDE, minimum-distortion embedding; GD, gamma delta T cells; MAIT, mucosal-associated invariant T cells; NKT, natural killer T cells; T_reg_, regulatory T cells; DN, double negative T cells; LLEC, long-lived effector cells.

To characterize the nonclassical T cell response to MCMV infection in the presence and absence of classical T cells, we performed multimodal single-cell RNA sequencing (scRNA-seq) in collaboration with ImmGen T (*42*) using C57BL/6 (B6) and K^b^D^b^-deficient mice. In addition, we used K^b^D^b−/−^Qa-1^−/−^ mice, which are deficient in both classical and Qa-1–restricted T cells. We performed TotalSeq/CITE-seq on splenic CD45^+^ CD3^+^ sorted T cells at both naïve state and following long-term MCMV infection (Fig. 4A and table S2). RNA expression and expression of 128 surface proteins (table S3) were measured in each single cell. For each sample, 274 to 1496 single cells passed quality control (QC) for surface protein expression and RNA expression (see Materials and Methods), totaling 9657 cells postfiltering. Cluster identities were assigned based on ImmGen T consortium analyses of all clusters across 96 single-cell experiments (*42*). All T cells from these experiments were isolated from the integrated consortium data, and unsupervised clustering revealed unique populations of nonclassical CD8^+^ T cells expanded in K^b^D^b−/−^ samples, as shown in the Uniform Manifold Approximation and Projection (UMAP) based on totalVI integration of RNA and surface protein expression data (Fig. 4, A and B).

In WT B6 mice, the proportion of cells in most T cell clusters increased following long-term infection, with the strongest enrichment observed in the activated CD8^+^, central memory CD8^+^, classical cytotoxic CD8^+^ (LLEC), classical effector CD8^+^, effector memory CD8^+^, and DN T #1 clusters (Fig. 4C). In K^b^D^b−/−^ mice, both exhausted CD8^+^ and “exhausted CD8^+^–low metabolism” clusters were enriched 5- and 12-fold, respectively, following MCMV infection as compared to naïve state (Fig. 4C), suggesting that MCMV-responsive Qa-1–restricted CD8^+^ T cells map to these clusters. The enrichment of these clusters was not observed in K^b^D^b−/−^Qa-1^−/−^ samples, indicating that Qa-1 is the main restricting element for the cells in these clusters (Fig. 4A). Notably, 63% of the total cells in the exhausted CD8^+^ T cell cluster following infection came from K^b^D^b−/−^ mice. In contrast to B6 and K^b^D^b−/−^ mice, K^b^D^b−/−^Qa-1^−/−^ mice did not display enrichment of any unique clusters and only displayed an increase of innate-like DN T #2 cluster (Fig. 4C). Together, these data suggest that the nonclassical CD8^+^ T cell-mediated response to MCMV infection is mostly driven by Qa-1–restricted CD8^+^ T cells.

### Nonclassical T cells display an exhausted phenotype during long-term MCMV infection

To determine the gene expression profiles of clusters that expanded in infected B6 and K^b^D^b−/−^ mice, we performed gene expression analysis of all CD8^+^ T cell clusters. Our analysis principally revealed genes involved in effector functions, memory, and exhaustion (Fig. 4D). To visualize an effector gene signature, a module score was produced using the expression of several key genes (*Cd44*, *Il7r*, *Cx3cr1*, *Klrg1*, and *Gzma*). Notably, the classical cytotoxic CD8^+^ (LLEC) and effector memory CD8^+^ clusters enriched in both B6 and K^b^D^b−/−^ mice following infection displayed the most robust expression of these genes (Fig. 4E). We observed that some common markers of exhaustion, including *Gzmk* and *Tigit*, were up-regulated in the exhausted CD8^+^ T cell cluster (Fig. 4D), which was primarily enriched following infection of K^b^D^b−/−^ mice. Therefore, a module score was also produced using expression of key genes associated with CD8^+^ T cell exhaustion (*Tox*, *Pdcd1*, *Lag3*, *Tigit*, and *Gzmk*), which were all specifically up-regulated in the nonclassical exhausted CD8^+^ clusters (Fig. 4F). To determine the transcriptional differences between nonclassical T cells that clustered with either the classical cytotoxic CD8^+^ (LLEC) or the exhausted CD8^+^ T cell subsets, all T cells from infected K^b^D^b−/−^ mice were isolated from the scRNA-seq dataset. Differential expression analysis of these two clusters (a stringent log_2_ fold change cutoff of 1.0 and a *P* value cutoff of 0.05) revealed that *Tox*, *Pdcd1*, *Lag3*, *Tigit*, and *Gzmk* were robustly expressed in the exhausted CD8^+^ T cell cluster (Fig. 4G). These data display that the effector genes expressed by classical T cells following MCMV infection are markedly different from those associated with the nonclassical T cell response, and that Qa-1–restricted CD8^+^ T cells acquired an exhausted phenotype following MCMV infection.

### Qa-1–restricted clonotypes expand following MCMV infection

**Fig. 5.**
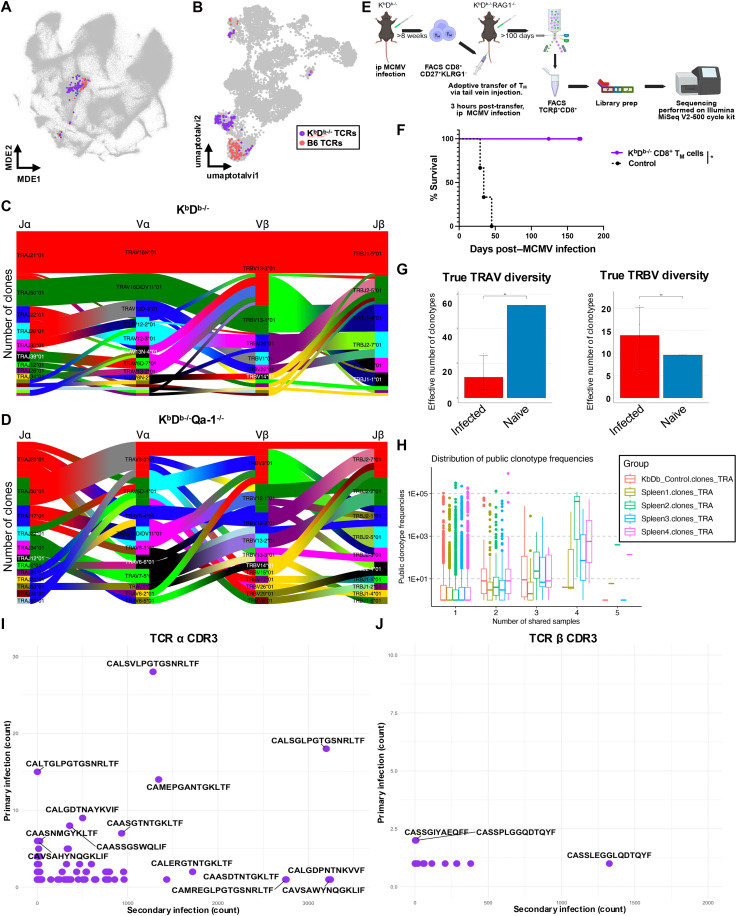
Qa-1–restricted clonotypes expand following MCMV infection. 2D-UMAP of cells expressing either the top six enriched clonotypes from K^b^D^b−/−^ (purple) or the top six clonotypes from B6 (red) mice on (**A**) all 96 ImmGen T single-cell experiments or (**B**) T cells from naïve and MCMV infected B6, K^b^D^b−/−^, and K^b^D^b−/−^Qa-1^−/−^ mice only. Plots created using totalVI integration and Seurat (v5). tcrdist3 analysis of the top 20 clonotypes expanded in (**C**) K^b^D^b−/−^ and (**D**) K^b^D^b−/−^Qa-1^−/−^ mice following chronic MCMV infection. Ribbon plots indicate TRAV and TRBV gene pairings for specific clonotypes, and the thickness of the ribbon directly correlates to the number of counts observed for that clonotype. Scalable vector graphic (SVG) plot created using tcrdist3 environment in Python (v3.10.10) and Jupyter notebook (v6.5.4). (**E**) Schematic depicting experimental design for bulk TCR-seq [Created in BioRender. Reilly, S. (2025); https://BioRender.com/2l67ypx]. (**F**) Survival of K^b^D^b−/−^RAG-1^−/−^ mice with (—) or without (– –) transfer of 5 × 10^4^ CD8^+^ T_M_ cells from long-term infected K^b^D^b−/−^ mice. Data are representative of two independent experiments, *n* = 3 to 5 per group. Statistical analysis used a Mantel-Cox test, and significant differences are denoted by *P < 0.05. (**G**) True diversity measure indicated by the effective number of clonotypes for TRAV and TRBV genes from infected versus naïve samples. (**H**) Clonotype distribution of public TCRα clonotypes across samples. Plots were created using Immunarch (v1.0.0). (**I**) TCRα and (**J**) TCRβ CDR3 sequences that expand following long-term primary MCMV infection (scTCR-seq) or long-term secondary infection (bulk TCR-seq) in K^b^D^b−/−^ mice. Dots colored purple depict clonotypes that overlap plotted on time point comparisons. Plots were created using ggplot2 (v3.5.2). ip, intraperitoneal.

In parallel with TotalSeq/CITE-seq analysis, we also performed TCR variable (TCR-V) sequencing (two-chains) at the single-cell level, using IMGT HighV-QUEST. Therefore, in addition to RNA and surface protein expression, TCRαβ paired clones were also determined. Productive TCRα and TCRβ sequences were available for 3828 (~40%) of the T cells analyzed. We identified six expanded clonotypes that were exclusively present in MCMV-infected K^b^D^b−/−^ mice (Table 1) and compared them to the top six observed in B6 mice. UMAP of these clonotypes revealed that these T cells belonged to either the exhausted CD8^+^ or the classical cytotoxic CD8^+^ (LLEC) T cell cluster, respectively (Fig. 5, A and B). While the top six clonotypes from K^b^D^b−/−^ infected mice constitute 21% of the exhausted CD8^+^ T cell cluster, K^b^D^b−/−^Qa-1^−/−^ mice did not display similar clonotype expansion. To investigate the V(D)J gene usage of the top clonotypes across samples, we used tcrdist3 (*43*) to visualize TCR gene usage from infected K^b^D^b−/−^ and K^b^D^b−/−^Qa-1^−/−^ mice. Paired clonotype abundance was represented with a visual ribbon plot generated using tcrdist3, whereby increased counts of a specific clonotype resulted in a thicker ribbon (Fig. 5, C and D). We observed that while gene usage was relatively diverse in K^b^D^b−/−^Qa-1^−/−^ samples, only a few Vα, and even fewer Vβ, genes occupied the repertoire space of the top clones in K^b^D^b−/−^ mice. Notably, no expanded clonotype from K^b^D^b−/−^ mice included *TRAV9D-3* nor *TRAV9N-3* gene usage, which has been shown to recognize Qa-1-FL9 (*44*), indicating that QFL T cells are not an expanded subset following long-term MCMV infection. This finding corroborated our previous finding that FL9 is not able to induce cytokine production from CD8^+^ T cells following reinfection of K^b^D^b−/−^ mice (Fig. 2). Furthermore, lack of clonal expansion in K^b^D^b−/−^Qa-1^−/−^ mice indicated that while other nonclassical T cells respond to MCMV infection, these responses are broad and marked by a diverse TCR repertoire.

**Table 1.** Top six TCR clonotypes expanded in K^b^D^b−/−^ mice following chronic MCMV infection. CD3^+^ T cells were sorted from the spleens of WT B6, K^b^D^b−/−^, and K^b^D^b−/−^Qa-1^−/−^ mice at naïve state and following long-term MCMV infection and subjected to scTCR-seq. Paired α and β gene usage and CDR3 sequences of the most enriched clonotypes found in nonclassical T cells following infection are listed.

TCR	α v gene	α j gene	CDR3α	β v gene	β j gene	CDR3β	Number of cells
#1	TRAV16N	TRAJ21	CAMREGLSNYNVLYF	TRBV13-3	TRBJ1-5	CASSADRQAPLF	42
#2	TRAV16D/DV11	TRAJ50	CAMREFLASSSFSKLVF	TRBV13-1	TRBJ1-4	CASSDVTASNERLFF	29
#3	TRAV12-3	TRAJ22	CALSDPPSGSWQLIF	TRBV26	TRBJ2-7	CASSPWGVEQYF	14
#4	TRAV12D-3 | TRAV12N-3	TRAJ28	CALTGLPGTGSNRLTF	TRBV1	TRBJ2-5	CTCSAAGGDTQYF	14
#5	TRAV6D-7 | TRAV6N-7	TRAJ39	CALGNNAGAKLTF	TRBV13-3	TRBJ1-1	CASSDRWGTEVFF	11
#6	TRAV12-2	TRAJ33	CALSHSNYQLIW	TRBV29	TRBJ2-7	CASSLGTGVEQYF	9

We next sought to determine whether the nonclassical clonotypes identified during primary infection would expand during secondary infection. As described above, we used an adoptive transfer model with RAG-1–deficient animals to enrich for protective nonclassical CD8^+^ T cells (Fig. 5E). Protective T cells from surviving transfer recipients were sorted and subjected to bulk TCR sequencing (TCR-seq) to examine the TCR repertoire (Fig. 5F). We observed limited TCRα diversity in infected samples as compared to a naïve K^b^D^b−/−^ control, represented by the effective number of clonotypes identified via Immunarch analysis of true diversity across samples (Fig. 5G). In contrast, TCRβ diversity was similar to that observed in naïve T cells. Moreover, we identified several public clonotypes within the TCRα repertoire that were shared across infected samples and displayed invariant gene usage of *TRAV12D-3 | TRAV12N-3* paired with *TRAJ28* (Table 2). These clonotypes displayed greater frequencies in infected samples as compared to the naïve control when shared across four or more samples (Fig. 5H). Notably, two complementarity-determining region 3 (CDR3) sequences (CALSGLPGTGSNRLTF; CALSVLPGTGSNRLTF) displayed a dominant expansion as they were present at a high frequency across infected samples, but not the naïve control, and differed by only one amino acid at P5 (Table 2). Two other public clonotypes also used *TRAV12D-3 | TRAV12N-3* paired with *TRAJ28*, corresponding to TCR clonotype #4 of the single-cell TCR-seq (scTCR-seq) experiment (Table 1). To highlight this overlap and others, we plotted the unpaired TCRα and TCRβ CDR3 sequences uncovered from scTCR-seq against those uncovered from our bulk TCR-seq approach. Remarkably, the two dominant CDR3α sequences observed following secondary infection were also the dominant CDR3α sequences observed following primary infection (Fig. 5I). These sequences are likely to be present on Qa-1–restricted T cells as K^b^D^b−/−^Qa-1^−/−^ mice did not display enrichment of these sequences. Although CDR3β sequences did not expand as robustly following primary infection, the observed overlap following secondary infection revealed shared motifs (CASSXXXXNQDTQYF) (Fig. 5J), indicating a preference for clonotype pairing with these motifs and a convergent immune response to common antigens. This invariant gene usage and convergent CDR3α sequence usage suggests that these public clonotypes recognize the same or similar antigen(s). Together, these data indicate that a few TCR clones that are potentially antigen-specific preferentially expand in Qa-1–restricted CD8^+^ T cells and that this population displays an exhausted phenotype during MCMV infection.

**Table 2.** MCMV protective nonclassical CD8^+^ T cells display TCRα public overlap. 5 × 10^4^ splenic CD8^+^ T_M_ (CD27^+^ KLRG1^−^) from long-term infected K^b^D^b−/−^ mice were transferred into simultaneously MCMV-infected K^b^D^b−/−^RAG-1^−/−^ mice. At ~100 days postinfection, 2 × 10^4^ splenic CD8^+^ T_M_ cells from K^b^D^b−/−^RAG-1^−/−^ mice were sorted and subjected to bulk TCR-seq (Fig. 5E). Gene usage, CDR3 sequence, and total counts of public TCRα clonotypes across all samples are indicated. High counts and single amino acid differences are bolded. NA, not applicable.

α v gene	α j gene	CDR3α	Samples	K^b^D^b−/−^ naïve	Spleen 1	Spleen 2	Spleen 3	Spleen 4
TRAV12D-3 | TRAV12N-3	TRAJ28	CALSGLPG**A**GSNRLTF	5	1	6	395	1	136
TRAV12D-3 | TRAV12N-3	TRAJ28	CALSGLPGTGSNRLTF	4	NA	**14157**	**121143**	72	**53376**
TRAV12D-3 | TRAV12N-3	TRAJ28	CALSGLPGT**R**SNRLTF	4	NA	3	12	1	6
TRAV12D-3 | TRAV12N-3	TRAJ28	CALS**V**LPGTGSNRLTF	4	4	4	**42811**	**18891**	NA

### Several HCMV peptides bind to HLA-E and share anchor residues with peptides that bind to Qa-1

Recently, the Birnbaum group generated a prediction algorithm to identify CMV peptides that are unlikely to bind CD94/NKG2A or CD94/NKG2C (*45*). Therefore, we used this list of over 60,000 CMV peptides predicted to subvert CD94/NKG2 receptor binding to generate an HCMV peptide library (table S4). The MCMV peptides M53, m59, m139, and m159 were used as input for FIMO motif discovery, as well as both leader sequences Qdm and VL9/UL40. In parallel, we used NetMHCpan EL to identify candidate peptide sequences predicted to be presented by HLA-E. Last, we tested whether peptide sequences were predicted to bind HLA-E*01:01, HLA-E*01:03, or Qa-1(b) using NetMHCpan BA. Priority was given to peptides with binding scores highest for HLA-E first, then Qa-1 (table S4).

**Fig. 6.**
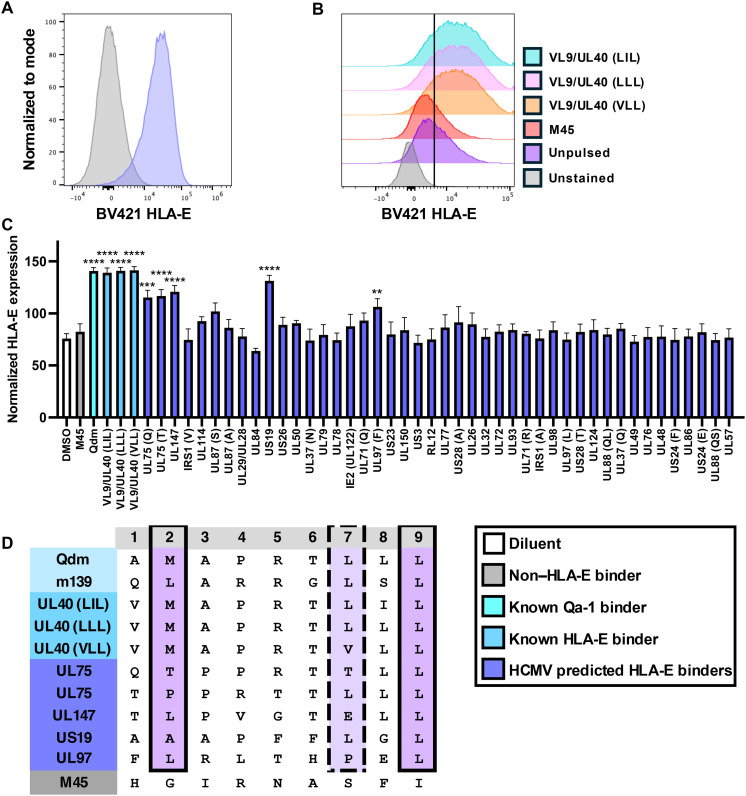
Several HCMV peptides bind to HLA-E and share anchor residues with peptides that bind to Qa-1. (**A**) Histogram depicting HLA-E surface expression on K562 cells (gray) or K562 cells transduced with HLA-E:01*03 (purple). (**B**) K562-HLA-E cells were pulsed with peptides and stained with an anti-HLA-E antibody. Histograms are representative of 11 independent experiments (gating strategy available in fig. S3). (**C**) Normalized HLA-E expression on the surface of K562-HLA-E cells following incubation with each indicated peptide or DMSO control. Peptides derived from the same HCMV protein are denoted by the first letter(s) of the peptide sequence in parentheses following the viral protein name. Data are pooled from 11 independent experiments, *n* = 4 to 11 per group. Data are presented as the means ± SEM. Statistical analysis used a one-way ANOVA, and significant differences are denoted by **P < 0.01; ***P < 0.001; ****P < 0.0001. (**D**) Peptide sequence schematic highlighting anchor positions between known HLA-E binders, Qa-1 binders, and HCMV peptides. Solid lines indicate the same or similar amino acid (AA) usage, while dotted lines indicate somewhat similar AA usage, based on biochemical properties.

To test whether HCMV peptides bind to HLA-E, we transduced the human HLA-E–negative cell line K562 with HLA-E (Fig. 6A). K562 cells are well known to be MHC-I null (*46*), and, therefore, expression of VL9 leader peptide is likely reduced (*47*). When K562-HLA-E was incubated with VL9/UL40 or Qdm peptides, we observed an approximately twofold increase of HLA-E expression at the cell surface (Fig. 6, B and C, and fig. S3) as reported by others using a different assay (*31*). Following incubation with candidate HCMV peptides, we found that several peptides induced a similar increase in HLA-E expression, demonstrating that these HCMV peptides bind to HLA-E (Fig. 6C). Similarly to MCMV binders, UL147 and UL97 peptides share typical hydrophobic anchors at residues P2 and P9 (Fig. 6D). Whereas AlphaFold3 in silico modeling revealed pLDDT confidence scores of over 80 for VL9/UL40 and most HCMV binders, UL75(T) and UL147 displayed lower overall confidence scores due to possible disorder or flexibility at P4 to P6 (fig. S3C). A recent study highlighted the critical role that P5 plays in the interaction with both CD94/NKG2A and the TCR (*48*). Although we observed a variation of amino acids at P5 in HLA-E binders (Fig. 6D), the peptide library was created using an algorithm designed to predict peptide sequences unlikely to bind CD94/NKG2A (*45*). Therefore, it is likely that this interaction is limited due to the inherent flexibility at P4 to P6 revealed by AlphaFold3 modeling. In addition, US19 also displayed possible disorder or flexibility at P5 to P6 (fig. S3C). Together, these data demonstrate that several HCMV-encoded peptides bind strongly to endogenous HLA-E.

### Some peptides that bind Qa-1 or HLA-E are cross-reactive

**Fig. 7.**
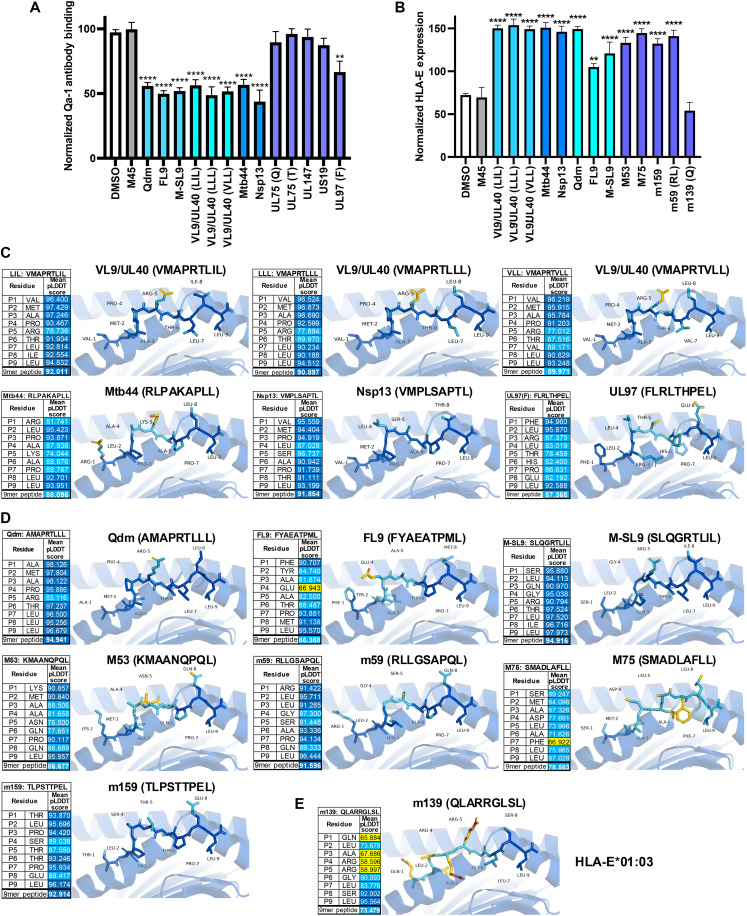
Some peptides that bind Qa-1 or HLA-E are cross-reactive. (**A**) Normalized Qa-1 antibody binding on the surface of P815 cells or (**B**) HLA-E expression on the surface of K562-HLA-E cells following incubation with each indicated peptide or DMSO control. Data are pooled from (A) 11 or (B) 5 independent experiments, *n* = 3 to 11 per condition. Data are presented as the means ± SEM. Statistical significance was determined using a one-way ANOVA, and significant differences are denoted by **P < 0.01 or ****P < 0.0001. (**C**) AlphaFold3 was used to model Qa-1-peptide complexes with HLA-E binding peptides that bound to Qa-1 in (A). (**D**) AlphaFold3 was used to model HLA-E-peptide complexes with Qa-1 binding peptides that bound to HLA-E in (B). (**E**) AlphaFold3 was used to model m139 in the binding groove of HLA-E:01*03. pLDDT score calculations are presented for each peptide, and the peptide residues are colored blue when the score was >90 (very high confidence), cyan when the score was 70 to 90 (confident prediction), yellow when the score was 50 to 70 (low confidence, indicating possible disorder or flexibility), or orange when the score was <50 (very low confidence).

Due to the structural similarities between the HLA-E and Qa-1 peptide-binding grooves (*49*) and the shared antigen specificity between the two molecules, we next investigated whether HCMV peptides could bind Qa-1 and whether MCMV peptides could bind HLA-E. We found that the VL9/UL40 derived peptides and UL97 were able to bind to Qa-1 (Fig. 7A). Similarly, the MCMV peptides M53, m59, M75, and m159, which bind Qa-1, were shown to bind HLA-E (Fig. 7B). In agreement with these data, AlphaFold3 modeling revealed strong confidence in the predicted structural interactions of these peptides within the peptide binding grooves of Qa-1 or HLA-E (Fig. 7, C and D). We found that peptides encoded by the severe acute respiratory syndrome coronavirus 2 (SARS-CoV-2) viral protein Nsp13 and *M. tuberculosis* protein Mtb44, which are presented by HLA-E (*50*, *51*), also bind to Qa-1 (Fig. 7A). Together, these findings demonstrate conservation of peptide presentation between the orthologs Qa-1 and HLA-E. We also found that the flu-derived M-SL9 binds to HLA-E (Fig. 7B), suggesting that this noncanonical cryptic peptide could have vaccine potential in humans.

## DISCUSSION

Our understanding of the role that nonclassical T cell responses play in antiviral immunity remains limited. While classical MHC-restricted CD8^+^ T cells have been extensively characterized in the context of viral infections, the contributions of nonclassical T cell populations are far less well defined. Emerging evidence suggests that these unconventional T cell responses may provide important layers of immune surveillance, particularly in immune-privileged tissues or during chronic and latent phases of infection (*52*). However, the mechanisms governing their activation, antigen specificity, and functional relevance in controlling viral replication or shaping long-term immunity are still poorly understood. Further investigation into these pathways could uncover previously unidentified strategies for enhancing immune protection, especially in contexts where classical pathways are evaded or suppressed by viral mechanisms. Our earlier work demonstrated that Qa-1–restricted CD8^+^ T cells can compensate in the absence of classical CD8^+^ effector responses during MCMV infection (*25*). Here, we extend these findings and identify Qa-1–restricted CD8^+^ T cell responses to multiple MCMV-derived peptides, demonstrating their functional significance. Specifically, using motif-based discovery and prediction pipelines, we identified M53, m59, m139, and m159 peptides with hydrophobic anchors at P2/P7/P9, consistent with established Qa-1/HLA-E binding preferences. AlphaFold3 structural modeling further underscored the high likelihood of stable peptide-MHC interactions. Functionally, ex vivo assays confirmed that m59, m139, and m159 peptides elicited Qa-1–restricted cytokine responses. In vivo, Qa-1 tetramer staining revealed robust expansion of m59- and m139-specific CD8^+^ T cells and accumulation in the salivary gland, the site of CMV latency. Geiger *et al.* recently demonstrated that QFL T cells expand during the early stages of MCMV infection, in response to virus-mediated down-regulation of ERAAP (*26*). However, in long-term infected animals, we did not observe a clonal expansion of the *TRAV9D-3 | TRAV9N-3* genes that define QFL T cells (*44*).

Consistent with the model of ongoing antigen exposure due to viral reactivation (*53*), we observed that Qa-1–restricted m139-specific CD8^+^ T cells expressed high levels of KLRG1, particularly in peripheral tissues (Fig. 3B). Single-cell transcriptomic profiling further revealed expansion of an effector-like T cell cluster expressing *Klrg1*, *Cx3cr1*, *Gzma*, and *S1pr5* in K^b^D^b−/−^ mice (Fig. 4E). These features parallel the inflationary CD8^+^ T cell phenotype previously described during chronic CMV infection (*54*). In contrast, a second expanded T cell cluster in K^b^D^b−/−^ mice expressed transcripts associated with exhaustion (i.e., *Tox*, *Pdcd1*, *Lag3*, *Tigit*, and *Gzmk*) (Fig. 4F). However, the TCR clonal expansion observed in this cluster suggest preserved or partial effector capacity. TCR repertoire analysis revealed a biased usage with convergent public TCRα CDR3 sequences sustained across both primary and secondary infections, indicating antigen-driven clonal selection and long-term persistence. Notably, this public overlap differed by only a single amino acid (Fig. 5I and Table 2), underscoring the fine specificity and potential for targeting by engineered therapeutics.

Our findings also highlight the translational relevance of Qa-1 and HLA-E as therapeutic targets. These nonclassical MHC-Ib molecules are orthologous and highly conserved, sharing 71% identity in the α1-α2 peptide-binding region (*49*). Extending these findings, we demonstrate cross-species epitope promiscuity, as the HCMV-derived peptide UL97 binds HLA-E and Qa-1. Moreover, we showed that four out of five MCMV peptides that bind Qa-1 also bind HLA-E, further supporting functional conservation across species (Fig. 7B). These observations align with accumulating evidence for HLA-E–restricted T cell responses in HCMV infection, including gpUL40 peptides (*55*, *56*), and their potential roles in viral control and transplantation scenarios. Notably, HCMV-induced HLA-E–restricted CD8^+^ T cells can persist long-term and even mediate alloreactive effects under certain contexts. This nonconventional T cell response to HCMV may be robust in some cases, as a recent study showed that HLA-E/UL40 tetramer^+^ αβ^+^ CD8^+^ T cells could be found in the peripheral blood of about one-third of the HCMV seropositive donors tested (*57*). Although it has been hypothesized that individuals may mount a CD8^+^ T cell response against CMV-derived UL40 when it mismatches their endogenous HLA-Ia leader peptide sequence (*58*), how the host immune system might distinguish between homeostatic HLA-E-VL9 and virally induced HLA-E-UL40 presentation is incompletely understood.

Our findings expand our understanding of nonclassical MHC-Ib immunity, suggesting that Qa-1/HLA-E–restricted responses supplement or rescue antiviral defenses when classical presentation is subverted. These results underscore that MHC-Ib antigen presentation via Qa-1/HLA-E integrates alternate peptide repertoires deriving from both pathogens and self, becoming particularly relevant during ERAAP down-regulation and MHC-I evasion. Vaccine and immunotherapeutic strategies could leverage Qa-1/HLA-E–binding epitopes, such as those from CMV, influenza (M-SL9), or even SARS-CoV-2, to elicit complementary CD8^+^ T cell responses that bypass viral immune evasion. Despite many HCMV vaccine candidates progressing through several phases of clinical trials, an HCMV vaccine has yet to be licensed (*59*). The cross-reactivity of CMV peptides with Qa-1 and HLA-E strengthens HLA-E as an intriguing target for therapeutic and vaccine development, particularly because HCMV and MCMV are closely related viruses. Moreover, the observed TCR clonal expansion suggests potential for designing TCR-mimetic immunotherapies or adoptive T cell transfer based on public clonotypes. In conclusion, our work uncovers a conserved and functionally significant nonclassical CD8^+^ T cell axis mediated by Qa-1/HLA-E, capable of recognizing viral epitopes, shaping adaptive immunity beyond classical MHC-I pathways, and offering promising targets for vaccine design and immunotherapy.

## MATERIALS AND METHODS

### Mice

K^b^D^b−/−^ mice were used as described previously, and K^b^D^b−/−^Qa-1^−/−^ mice were generated via CRISPR-Cas9, as described in (*25*). C57BL/6J (B6; strain no. 000664, RRID:IMSR_JAX:000664) mice were purchased from the Jackson Laboratory and then maintained in-house. None of the experiments within these studies were blinded or randomized. Both age- and sex-matched mice were used for this study.

### Institutional Animal Care and Use Committee statement

All experiments were carried out in strict accordance with the recommendations in the Guide for the Care and Use of Laboratory Animals, as defined by the National Institutes of Health (NIH; PHS Assurance no. D16-00183). Animal protocol no. 23-08-0002 was reviewed and approved by the Institutional Animal Care and Use Committee of Brown University. All animals were housed in a centralized and AAALAC (Association for Assessment and Accreditation of Laboratory Animal Care International)-accredited research animal facility that is fully staffed with trained husbandry, technical, and veterinary personnel.

### Cell lines and culture conditions

P815 [American Type Culture Collection (ATCC) no. TIB-64, RRID:CVCL_2154] and K562 (ATCC no. CCL-243, RRID:CVCL_0004) cells were cultured in Dulbecco’s modified Eagle’s medium (DMEM)/high glucose with l-glutamine (4 mM), glucose (4500 mg/liter), and sodium pyruvate (Thermo Fisher Scientific no. SH30243.01). In all cases, media contained 8% fetal bovine serum (Atlanta Biologics no. S11550 or Gibco no. 10437-028), penicillin and streptomycin and glutamine (100 U/ml; Gibco no. 10378-016), and 48 μM β-mercaptoethanol (Gibco no. 21985-023). Cells were cultured at 37°C in 5% CO_2_. The K562-HLA-E cell line was made by infection with an HLA-E–containing lentivirus.

### Generation of HLA-E lentivirus and K562-HLA-E

HLA-E*01:03 open reading frame was cloned into a pGenlenti vector by GenScript. Brown University Lentivirus Construct Core generated HLA-E_pGenlenti lentivirus with the plasmid, and viral particles were transduced into the K562 cell line. Confirmation of successful lentiviral transduction was confirmed with drug resistance to puromycin. HLA-E surface expression was confirmed by flow cytometry.

### Preparation of virus stocks

MCMV-RVG102 was a gift from J. Hamilton (Duke University) and expressed recombinant enhanced green fluorescent protein under the immediate early-1 (IE1) promoter (*60*). Stocks were prepared in vivo from SMG homogenate, as previously described (*61*). MCMV m139*stop* was a gift from W. Brune (Leibniz Institute of Virology) and contains a stop codon at position 32 of m139 (*40*). Stocks were prepared in vitro using the mouse embryonic fibroblast (MEF) cell line NIH/3T3 (ATCC no. CRL-1658, RRID:CVCL_0594). Briefly, 3T3 cells grown in 10% DMEM-serum at 37°C with 5% CO_2_ were infected with virus, and viral particles were collected from the supernatant and filtered. Viral titer plaque-forming units (PFU) were determined via standard plaque assay using MEF cells. For plaque assays, MEF cells were grown in 10% DMEM-serum at 37°C with 5% CO_2_ and then infected for 6 days in a methylcellulose overlay, fixed with 10% formalin, and stained with crystal violet to visualize plaques.

### Generation of peptide libraries

To generate a list MCMV peptides, we first used FIMO, a software tool available through the Meme Suite (*62*), which can generate a list of peptides from a given genome that are likely to share a motif with an input protein sequence. Following FIMO output, the MHC-I binding prediction tool available from IEDB-AR (*34*) was used to predict whether each peptide sequence was likely to bind to Qa-1 or HLA-E (separate analyses). Last, viral proteins that encoded the top peptides were analyzed using PROSPER (*35*) to predict whether the peptide contained a protease cleavage site within it. Peptides that were predicted to exist in vivo were organized by their predicted binding strength to Qa-1 first, then HLA-E to generate the final MCMV peptide library.

The HCMV peptide library was generated by using the list of CMV peptides described in (*45*). Using FIMO, Qa-1 binding peptides were used as input for motif scanning of this HCMV peptide list to identify peptides that shared a motif with one or more Qa-1 binding peptides. The IEDB-AR MHC-I binding prediction tool was then used to rank peptides by their predicted binding strength to HLA-E first, then Qa-1.

### Synthetic peptides

Synthetic peptides outlined in tables S1 and S4 were obtained at >85% purity from GenScript and dissolved in dimethyl sulfoxide (DMSO) at a stock concentration of 10 mg/ml.

### Peptide binding assays

For Qa-1 peptide binding assays, 2.5 × 10^5^ P815 cells were incubated with 20 μM of synthetic peptide at 32°C for 20 min in a V-bottomed plate. For HLA-E peptide binding assays, 1 × 10^5^ K562-HLA-E cells were incubated with 100 μM of synthetic peptide at 29°C overnight. Immediately following incubation, cells were stained with either a Qa-1 or an HLA-E antibody.

### Viral infection protocols

MCMV-RVG102 infections were performed with 5 × 10^4^ PFU intraperitoneally, and MCMV m139*stop* infections were performed with 5 × 10^5^ PFU intraperitoneally. Mice were considered long-term infected at >8 weeks post–MCMV infection. Following long-term infection, reinfection experiments used the same virus as primary infection. A mouse was only excluded from analysis if determined to be aberrantly infected, based on lack of KLRG1 up-regulation in the animal. For some experiments, blood was collected from live mice, processed, and stained for infection markers, including KLRG1 and M45 tetramer.

### Lymphocyte isolation

Mice were humanely euthanized with isoflurane and cardiac puncture or cervical dislocation before organ removal. Spleens were manually dissociated using the hard end of a syringe plunger in 150 mM NH_4_Cl for 10 min, filtered through nylon mesh, and washed twice with 1% phosphate-buffered saline (PBS)–serum. Live cell counts were obtained using either a hemocytometer with trypan blue exclusion or a Countess 3 cell counter. SMGs were processed manually to remove the sublingual glands, parotid glands, and lymph nodes. The isolated SMGs were digested in 2 ml of digestion solution (DMEM) with Liberase DL (16.7 μg/ml; Sigma-Aldrich no. 05 466 202 001) and deoxyribonuclease I (0.3 U/ml; Sigma-Aldrich no. D7291-2MG) on the heart01.01 program of a GentleMACS and incubated at 37°C for 10 min. This process was repeated for a total of two dissociations and two incubations. After digestions, the SMG samples were filtered through nylon mesh and washed once with 1% PBS-serum before a Lympholyte-M (Zageno no. CL5035) gradient underlay. Lymphocytes were harvested from the gradient interface and washed once in 1% PBS-serum. All primary cell samples were maintained at 0° to 4°C during preparation (except as otherwise specified) until analysis.

### Secondary MCMV challenge

Following lymphocyte isolation, samples were enriched for CD8^+^ cells using an OctoMACS and CD8α MicroBeads (Miltenyi Biotech no. 130-117-044). For SMG-derived transfer experiments, 50,000 to 150,000 CD8^+^ T cells pooled from six long-term infected K^b^D^b−/−^ SMGs were transferred per recipient. For splenic-derived transfer experiments, 50,000 CD8^+^ memory T (T_M_) cells (CD8^+^ KLRG1^−^CD27^+^) sorted from long-term infected K^b^D^b−/−^ spleens were transferred per recipient. Cells were injected intravenously into K^b^D^b−/−^RAG-1^−/−^ mice, and recipients were infected with MCMV 3 hours postinjection for all transfer experiments.

### Ex vivo CD8^+^ T cell stimulation

Splenocytes were isolated from individual K^b^D^b−/−^ and K^b^D^b−/−^Qa-1^−/−^ mice. One to two million cells per well were plated in duplicate in flat-bottomed 96-well tissue culture plates and stimulated in the presence of synthetic peptides (100 μM final concentration for single peptides and 40 μM final concentration for pooled peptides) or added to wells coated with anti-mouse CD3ε (BioLegend no. 100301, RRID:AB_312666) and CD28 antibodies (BioLegend no. 102101, RRID:AB_312866). DMSO (0.2%) was included as an additional control to account for the increase in DMSO concentration present under the pooled peptides condition. Stimulated cells were incubated for 6 hours at 37°C in 10% DMEM-serum + GolgiPlug Protein Transport Inhibitor (BD Biosciences no. 554724), per the manufacturer’s instructions. Following incubation, duplicate samples were pooled and stained for intracellular IFN-γ, TNF-α, and IL-2 production. In some cases, anti-CD107α (2.5 μg/ml) was included along with GolgiPlug during the 6-hour incubation.

### Antibodies and flow cytometry

For experiments using mouse cells, single cell suspensions were stained in 1% PBS-serum containing Fc block (0.5 μg/ml) (clone 2.4G2; either produced in-house or Bio X Cell no. BE0307, RRID:AB_2736987) and cell surface antibodies for 20 min on ice in the dark. When staining for Qa-1, cells were stained with Biotin Mouse Anti-Mouse Qa-1b (BD Biosciences no. 559829, RRID:AB_397345) for 15 min on ice in the dark, followed by a second 15-min incubation on ice in the dark with Allophycocyanin (APC) Streptavidin (Invitrogen no. 17-4317-82) before analysis. Staining with APC Qa-1/Qdm, APC Qa-1/m59, and APC Qa-1/m139 (NIH Tetramer Core Facility, RRID:SCR_026557) was performed for 20 min at room temperature in the dark. Samples stained without tetramer were used as a gating control. For intracellular staining, cells were fixed using Cytofix/Cytoperm (BD Biosciences no. 554722) for 30 min and stained in Perm/Wash Buffer (BD Biosciences no. 554723) for 20 min on ice in the dark. Samples were run on either a four-laser (16UV-16V-14B-8R) Cytek Aurora (Cytek Biosciences) or five-laser FACSAria III (BD Biosciences) and analyzed using FlowJo (Tree Star Inc., RRID:SCR_008520). Antibody titration was performed on either primary mouse splenocytes or cell lines.

The mAbs used for flow cytometry were purchased from BioLegend, eBioscience, BD Biosciences, or Thermo Fisher Scientific. Brilliant Stain Buffer (BD Biosciences no. 563794) was included when two or more Brilliant Violet antibodies were included in the staining cocktail. Mouse antibodies used include fluorescein isothiocyanate (FITC) Rat Anti-Mouse CD107a (LAMP-1) (BD Biosciences no. 553793, RRID:AB_395057), BV421 Rat Anti-Mouse CD127 (BioLegend no. 135023, RRID:AB_10897948), peridinin-chlorophyll-protein (PerCP)-eFluor710 Rat Anti-Mouse CD127 (eBioscience no. 46-1273-82, RRID:AB_2573710), BV605 Hamster Anti-Mouse CD27 (BD Biosciences no. 563365, RRID:AB_2738160), phycoerythrin (PE) Rat Anti-Mouse CD27 (eBioscience no. 12-0271-82, RRID:AB_465614), PerCP-eFluor710 Hamster Anti-Mouse CD27 (eBioscience no. 46-0271-82, RRID: AB_1834447), BUV395 Hamster Anti-Mouse CD3e (BD Biosciences no. 563565, RRID: AB_2738278), BV570 Rat Anti-Mouse CD4 (BioLegend no. 100542, RRID:AB_2563051), BV650 Rat Anti-Mouse CD4 (BioLegend no. 100469, RRID: AB_2783035), APC-eFluor780 Rat Anti-Mouse CD45 (Invitrogen no. 47-0451-82, RRID:AB_1548781), SuperBright600 Rat Anti-Mouse CD45 (Invitrogen no. 63-0451-82, RRID:AB_2637149), FITC Mouse Anti-Mouse CD45.1 (eBioscience no. 11-0453-82, RRID:AB_465058), PE Mouse Anti-Mouse CD45.1 (eBioscience no. 12-0453-83, RRID:AB_465062), FITC Mouse Anti-Mouse CD45.2 (eBioscience no. 11-0454-85, RRID:AB_465062), PE Mouse Anti-Mouse CD45.2 (eBioscience no. 12-0454-83, RRID: AB_465679), BV570 Rat Anti-Mouse CD45 (BioLegend no. 103136, RRID: AB_2562612), BV711 Rat Anti-Mouse CD62L (BioLegend no. 104445, RRID: AB_2564215), eFluor450 Rat Anti-Mouse CD8α (eBioscience no. 48-0081-82, RRID:AB_1272198), BV605 Rat Anti-Mouse CD8α (BioLegend no. 100744, RRID: AB_2562609), PE-Texas Red Rat Anti-Mouse CD8α (Thermo Fisher Scientific no. MCD0817, RRID: AB_10374589), APC Mouse Anti-Mouse CX3CR1 (BioLegend no. 149008, RRID:AB_2564492), APC Rat Anti-Mouse IFN-γ (eBioscience no. 17-7311-81, RRID: AB_469503), eFluor450 Rat Anti-Mouse IFN-γ (eBioscience no. 48-7311-82, RRID:AB_1834366), PE Rat Anti-Mouse IL-2 (BioLegend no. 503807, RRID:AB_315301), BV421 Hamster Anti-Mouse KLRG1 (BD Biosciences no. 566284, RRID: AB_2739658), PE-Cyanine7 Hamster Anti-Mouse KLRG1 (eBioscience no. 25-5893-82, RRID: AB_1518768), BV785 Mouse Anti-Mouse NK1.1 (BioLegend no. 108749, RRID: AB_2564304), FITC Rat Anti-Mouse NKG2A/C/E (Invitrogen no. 11-5896-82, RRID: AB_465305), PerCP-eFluor710 Rat Anti-Mouse NKG2A/C/E (eBioscience no. 46-5896-82, RRID:AB_10853352), PE Rat Anti-Mouse CD279 (PD-1) (BioLegend no. 135205, RRID: AB_1877232), BV421 Rat Anti-Mouse CD279 (PD-1) (BioLegend no. 135218, RRID: AB_2561447), BUV496 Hamster Anti-Mouse TCRβ (BD Biosciences no. 749915, RRID: AB_2874154), BV510 Hamster Anti-Mouse TCRβ (BioLegend no. 109234, RRID: AB_2562350), FITC Rat Anti-Mouse TCR Vα 3.2b,c (BD Biosciences no. 553219, RRID:AB_394715), PerCP-eFluor710 Rat Anti-Mouse TCR Vα 3.2 (eBioscience no. 46-5799-82, RRID:AB_11150254), PE-Cyanine5 Rat Anti-Mouse TIGIT (BioLegend no. 622207, RRID:AB_3106166), APC Rat Anti-Mouse TNF-α (eBioscience no. 17-7321-82, RRID:AB_469508), and AlexaFluor657 Rat Anti-Mouse TNF-α (BioLegend no. 506314, RRID:AB_493330). Human antibodies used include Human TruStain FcX (Fc Receptor Blocking Solution) (BioLegend no. 422302, RRID:AB_2818986) and BV421 Mouse Anti-Human HLA-E (BioLegend no. 342612, RRID:AB_2721525).

### Single-cell RNA, TCR, and TotalSeq-C sequencing—Sample preparation

#### Flow cytometry and HT antibody staining

Single-cell lymphocyte suspensions were isolated as described above and stained with anti-CD45-APC (BioLegend no. 103112, RRID:AB_312977), anti-CD3ε-PE (BioLegend no. 100308, RRID:AB_312673), and different TotalSeq-C Anti-Mouse Hashtags (table S2; TotalSeq-C Anti-Mouse Hashtags 1–10 BioLegend no. 155861–155879, RRID:AB_2800693–97 and AB_2819910–14) in staining buffer (DMEM, 2% FBS, and 10 mM Hepes) for 20 min at 4°C in the dark. Spleen standard cell suspensions were stained under the same conditions but used anti-CD45-FITC (BioLegend no. 109806, RRID:AB_313443) to distinguish it from the other samples once pooled. Each sample was stained with a single hashtag, which allows for the downstream assignment of individual 10x cell barcodes to their source samples (*63*).

#### Cell sorting

For each sample, 25,000 to 40,000 4′,6-diamidino-2-phenylindole (DAPI)^−^ CD45^+^ CD3^+^ T cells were sorted on the FACSAria III and pooled in a single collection tube at 4°C. DAPI was added just before the sort to exclude dead cells.

#### ImmGenT TotalSeq-C custom mouse panel staining

The pooled single-cell suspension was stained with the ImmGenT TotalSeq-C custom mouse panel, containing 128 antibodies (table S2; BioLegend no. 900004815) and FcBlock (Bio X Cell no. BE0307, RRID:AB_2736987). Because 500,000 cells were required for staining, unstained splenocytes were spiked in to reach a total of 500,000 cells.

#### Second sort

Cells were subsequently sorted a second time with the addition of DAPI to select for live CD45^+^ CD3^+^ T cells. A total of 45,000 CD45-APC^+^ sample cells and 5000 CD45-FITC^+^ spleen standard cells were sorted into a single collection tube.

### Single-cell RNA, TCR, and TotalSeq-C sequencing—Library preparation

The collected cells were pooled, and scRNA-seq was performed using the 10x Genomics 5′ v2 with Feature Barcoding for Cell Surface Protein and Immune Receptor Mapping following the manufacturer’s instructions (CG000330). After cDNA amplification, the smaller fragments containing the TotalSeq-C–derived cDNA were purified and saved for Feature Barcode library construction. The larger fragments containing transcript-derived cDNA were saved for TCR and Gene Expression library construction. For both cDNA portions, library size was measured by an Agilent Bioanalyzer 2100 High Sensitivity DNA Assay and quantified using a Qubit dsDNA HS Assay Kit on a Qubit 4.0 Fluorometer.

#### TCRαβ amplification and library construction

TCR cDNA was amplified from the transcript-derived cDNA by nested polymerase chain reaction (PCR) according to the manufacturer’s protocol. The TCR libraries were then subjected to enzymatic fragmentation, ligation, and indexing with unique Dual Index TT Set A (10x part no. 3000431) index sequences, TT-A9 and TT-A10, with an index PCR (eight cycles).

#### RNA library construction

Transcript-derived cDNA was processed into the RNA libraries. Following enzymatic fragmentation and size selection, purified cDNA was ligated to an Illumina R2 sequence and indexed with unique Dual Index TT Set A index sequences, TT-H6 and TT-H7, with an index PCR (14 cycles).

#### TotalSeq-C library construction

TotalSeq-C derived cDNA was processed into the Feature Barcode libraries. The cDNA was indexed with unique Dual Index TN Set A (10x part no. 3000510) index sequences, TN-C8 and TN-C9, with an index PCR (eight cycles).

#### Sequencing of libraries

The three libraries were pooled together based on molarity with relative proportions 47.5% RNA, 47.5% Feature Barcode, and 5% TCR. This pool was then sequenced on an Illumina NovaSeq S2, 100c. The sequencing configuration followed 10x specifications, namely, 26 cycles in Read 1, 10 in Index 1, 10 in Index 2, and 90 in Read 2.

### Single-cell RNA, TCR, and TotalSeq-C sequencing—Data processing

#### Count matrices

Gene and TotalSeq-C antibody (surface protein panel and hashtags) counts were obtained by aligning reads to mm10(GRCm38) and the M25(GRCm38.p6). GeneCode annotation (GRCm38.p6) of the mouse genome and the DNA barcode for the TotalSeq-C panel (table S3) using CellRanger software (RRID:SCR_017344) v7.1.0 (10x Genomics) with default parameters. Cells were distinguished from droplets with high RNA and TotalSeq-C counts using inflection points on total count curve (barcodeRanks function in the DropletUtils package).

#### Sample demultiplexing

Sample demultiplexing used the Hashtag counts and the HTODemux from the Seurat package (RRID:SCR_016341) v4.1. Doublets (droplets with two hashtags) were excluded, and cells were assigned to the maximum hashtag signal if it had at least 10 counts and more than twice the signal for the second most abundant hashtag. The hashtag count data were also analyzed by t-distributed stochastic neighbor embedding (t-SNE) for a visual check (clear separated clusters for each hashtag). All single cells from the gene count matrix were matched unambiguously to a single hashtag (and, therefore, their original sample).

#### QC and batch correction

Cells matching at least one of the following criteria were excluded: fewer than 500 RNA counts; dead cells with more than 10% of counts mapping to mitochondrial genes; less than 500 TotalSeq-C counts; and cells positive for two isotype controls (nonspecific binding of antibodies).

#### Clustering and dimensionality reduction

Data from the two technical replicates were merged and normalized using centered log ratio. The top 2000 variable genes were found using the FindVariableFeatures function in Seurat (v4.2.0) and the variance-stabilizing transformation (VST method). Principal components analysis was performed using the top 2000 genes. The top principal components (PC) explaining 80% of the total variance were used for two-dimensional (2D) reduction using UMAP. Clustering was performed on the same PCs using the FindClusters() function in Seurat.

#### Differential expression

To determine differentially expressed genes, FindMarkers() within Seurat was used. AddModuleScore() was used to visualize aggregated expression of a set of genes. Volcano plots were created using EnhancedVolcano (v1.14.0).

#### TCRαβ analyses

TCRαβ contigs for each single cell were obtained by aligning reads to reference genes (refdata-cellranger-vdj-GRCm38-alts-ensembl-7.0.0) using CellRanger software (v7.1.0) with default parameters. Immunarch (v1.0.0) was used to analyze TCRαβ repertoires and gene usage. TCR gene usage analyses were also performed with tcrdist3 using Python (v3.10.10) and Jupyter notebook (v6.5.4).

### Bulk TCR-seq—Sample preparation, library preparation, and data processing

#### Flow cytometry and antibody staining

Single-cell lymphocyte suspensions were isolated from K^b^D^b−/−^RAG-1^−/−^ recipients as described above and stained with anti-TCRβ-BV510 (BioLegend no. 109234, RRID:AB_2562350) and anti-CD8α-eFluor450 (eBioscience no. 48-0081-82, RRID:AB_1272198) in staining buffer (DMEM, 2% FBS, and 10 mM Hepes) for 20 min at 4°C in the dark.

#### Cell sorting

For each sample, 20,000 TCRβ^+^ CD8α^+^ T cells were sorted on the FACSAria III and pooled in a single collection tube containing RNAprotect Cell Reagent (QIAGEN no. 76526) at 4°C. Following collection, samples were stored at −20°C and shipped to iRepertoire, Inc. (Huntsville, AL) on dry ice. The collected cells were pooled and split into two technical replicates for all subsequent steps.

#### Library preparation

iRepertoire, Inc. performed library preparation in accordance with mouse RepSeq^+^ service, which included amplification of both TCRα and TCRβ chains using dimer-avoided multiplex PCR (dam-PCR) technology. Following library preparation, samples were indexed and pooled, and sequencing was performed on an Illumina MiSeq with a V2-500 cycle kit with a read depth of 1 million reads per sample.

#### Data processing

Paired-end FastQC files from independent replicates were concatenated and used as input for the analysis. The alignment and assembly of TCR RNA-seq reads and the exporting of clonotypes were conducted using MiXCR (*64*). Immunarch (v1.0.0) was used to analyze TCRαβ repertoires and visualize basic sample statistics.

### AlphaFold3 modeling

Predicted 3D protein structures of Qa-1/HLA-E peptide docking sites with peptide loaded were generated using the AlphaFold3 public web server (accessed May 2025; RRID:SCR_021709). Inputs included α1-3, β2-microglobulin, and peptide sequences. Five models were generated per complex, ranked by AlphaFold3 ranking score (*37*). The top-ranking model was selected, and PyMOL software (RRID: SCR_000305) v3.1.1 was used to visualize the 3D structures. For each AlphaFold3 model, pLDDT scores were embedded in the output file and each residue score (P1 to P9) was calculated by averaging the individual pLDDT scores per atom. For the nonamer (9mer) peptide score, the mean of the raw atomic pLDDT values for all nine residues was calculated.

### Statistical analysis

Analyses for determining statistical significance were performed using Prism 10.0 (GraphPad Software, Inc., RRID: SCR_002798). Unpaired two-tailed Student’s *t* tests were used to compare two individual groups, whereas analysis of variance (ANOVA) with multiple comparisons test was used when more than two groups were compared. Graphs represent the mean, and error bars indicate standard error of the mean (SEM). The number of animals per sample size and total experimental replicates are noted in each figure legend. *P < 0.05; **P < 0.01; ***P < 0.0001; and ****P < 0.00001.
