# Supplementary material for: Unconventional CD8+ T cell surveillance of cytomegalovirus via Qa-1/HLA-E–restricted epitope recognition
Source: Sci Adv. 2025 Dec 19;11(51):eaea8707. doi: 10.1126/sciadv.aea8707 (PMC12716397; doi:10.1126/sciadv.aea8707)
Supplement: Supplementary file 1 — Figs. S1 to S3 Tables S1 to S4 Legends for files S1 to S15 [file sciadv.aea8707_sm.pdf]

Science Advances
AAAS

Supplementary Materials for

Unconventional CD8$^+$ T cell surveillance of cytomegalovirus via Qa-1/ HLA-E–restricted epitope recognition

Shanelle P. Reilly *et al.*

Corresponding author: Laurent Brossay, laurent_brossay@brown.edu

Sci. Adv. **11**, eaea8707 (2025)
DOI: 10.1126/sciadv.aea8707

The PDF file includes:

Figs. S1 to S3
Tables S1 to S4
Legends for files S1 to S15

Other Supplementary Material for this manuscript includes the following:

Files S1 to S15

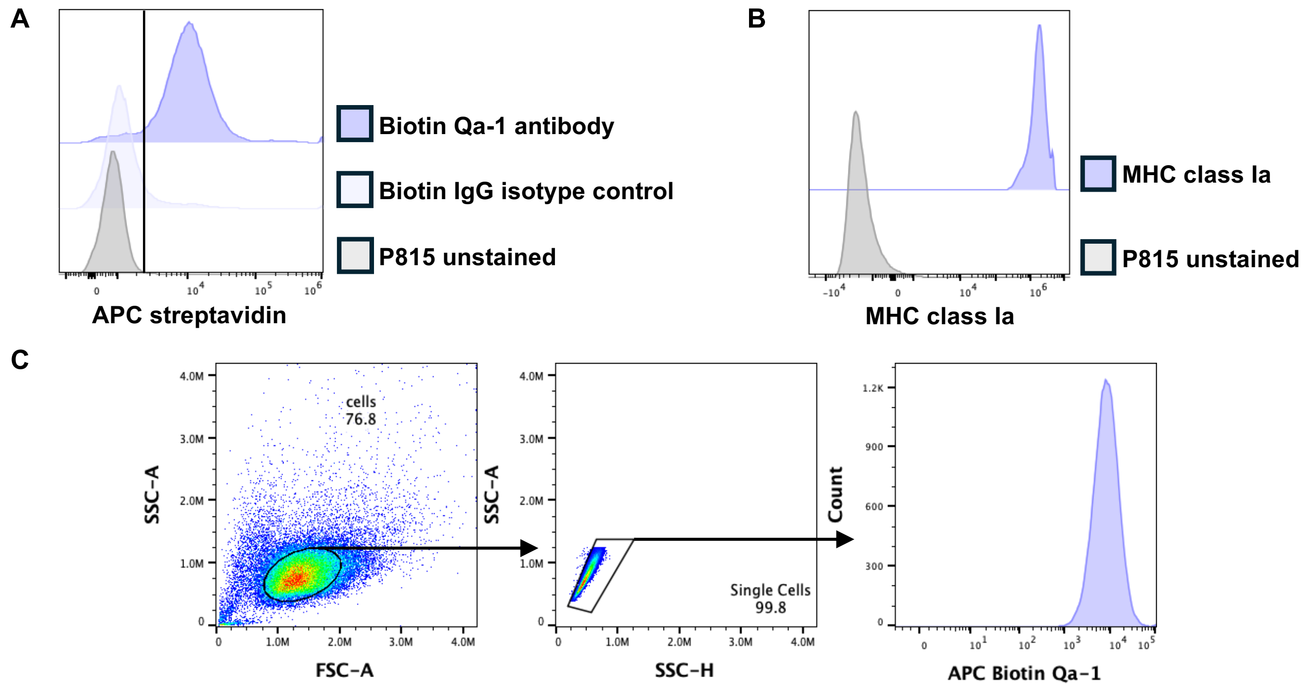

D

Qdm: AMAPRTLLL		
Residue		Mean pLDDT Score
P1	ALA	96.962
P2	MET	96.741
P3	ALA	95.614
P4	PRO	90.376
P5	ARG	75.996
P6	THR	88.193
P7	LEU	90.011
P8	LEU	89.921
P9	LEU	94.837
9mer Peptide		89.907

Qdm (AMAPRTLLL)

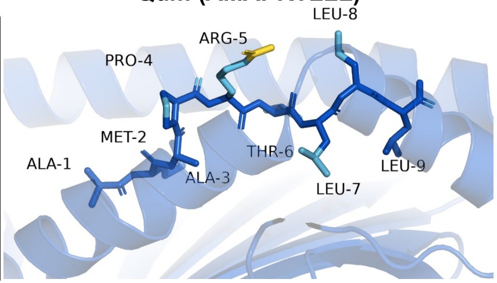

FL9: FYAEATPML		
Residue		Mean pLDDT Score
P1	PHE	93.855
P2	TYR	96.688
P3	ALA	93.064
P4	GLU	76.850
P5	ALA	81.996
P6	THR	76.150
P7	PRO	80.086
P8	MET	83.410
P9	LEU	91.173
9mer Peptide		86.624

FL9 (FYAEATPML)

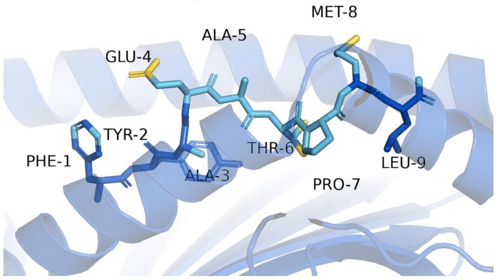

M-SL9: SLQGRTLIL		
Residue		Mean pLDDT Score
P1	SER	96.393
P2	LEU	96.421
P3	GLN	88.190
P4	GLY	90.245
P5	ARG	81.995
P6	THR	92.296
P7	LEU	93.001
P8	ILE	92.983
P9	LEU	95.927
9mer Peptide		91.481

M-SL9 (SLQGRTLIL)

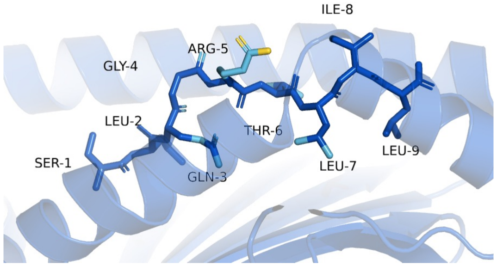

M53: KMAANQPQL		
Residue		Mean pLDDT Score
P1	LYS	88.423
P2	MET	96.251
P3	ALA	94.896
P4	ALA	87.182
P5	ASN	81.935
P6	GLN	75.372
P7	PRO	87.620
P8	GLN	80.907
P9	LEU	92.993
9mer Peptide		86.790

M53 (KMAANQPQL)

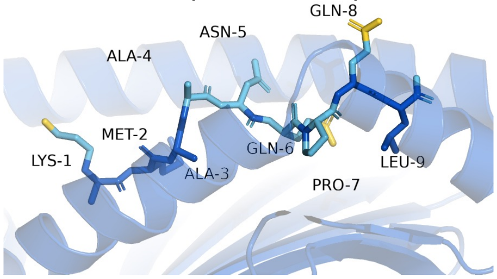

m59: RLLGSAPQL		
Residue		Mean pLDDT Score
P1	ARG	76.365
P2	LEU	92.484
P3	LEU	84.213
P4	GLY	81.435
P5	SER	78.128
P6	ALA	86.122
P7	PRO	86.839
P8	GLN	82.368
P9	LEU	93.776
9mer Peptide		84.655

m59 (RLLGSAPQL)

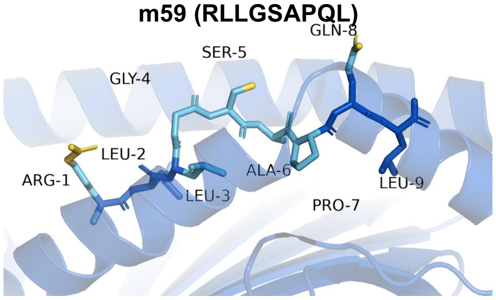

M75: SMADLAFLL		
Residue		Mean pLDDT Score
P1	SER	94.480
P2	MET	94.975
P3	ALA	94.154
P4	ASP	82.006
P5	LEU	73.433
P6	ALA	84.206
P7	PHE	84.525
P8	LEU	92.795
P9	LEU	93.630
9mer Peptide		87.894

M75 (SMADLAFLL)

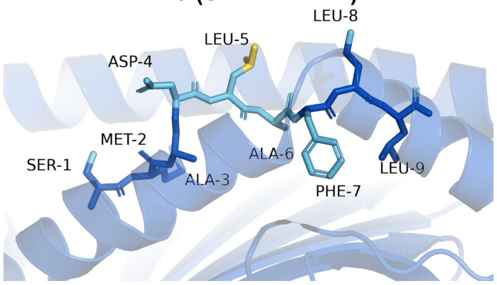

m139: QLARRGLSL		
Residue		Mean pLDDT Score
P1	GLN	84.191
P2	LEU	93.266
P3	ALA	92.462
P4	ARG	66.173
P5	ARG	71.013
P6	GLY	89.998
P7	LEU	92.303
P8	SER	92.228
P9	LEU	93.533
9mer Peptide		84.067

m139 (QLARRGLSL)

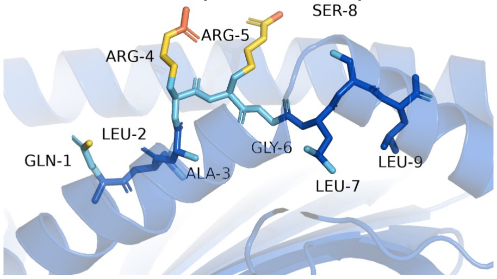

m159: TLPSTTPEL		
Residue		Mean pLDDT Score
P1	THR	92.539
P2	LEU	95.703
P3	PRO	94.246
P4	SER	86.928
P5	THR	82.840
P6	THR	85.804
P7	PRO	89.313
P8	GLU	84.309
P9	LEU	93.536
9mer Peptide		89.567

m159 (TLPSTTPEL)

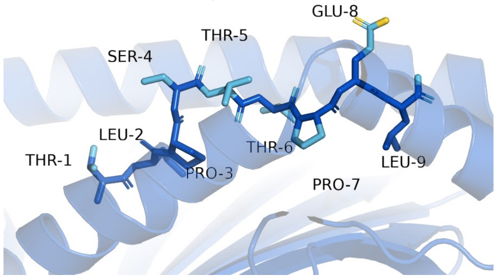

Supplemental Figure 1. (**A**) P815 cells were stained with either Qa-1 antibody or isotype control. Secondary staining was performed with streptavidin conjugated to APC. (**B**) P815 cells were stained with an MHC class Ia antibody. (**C**) Gating strategy for Qa-1 binding assay performed on P815 cell line. (**D**) Alphafold3 was used to model Qa-1-peptide complexes with MCMV peptides that bound to Qa-1 in **Fig. 1C**. pLDDT score calculations are presented for each peptide and the peptide residues are colored blue when the score was >90 (very high confidence), cyan when the score was 70-90 (confident prediction), yellow when the score was 50-70 (low confidence, indicating possible disorder or flexibility), or orange when the score was <50 (very low confidence).

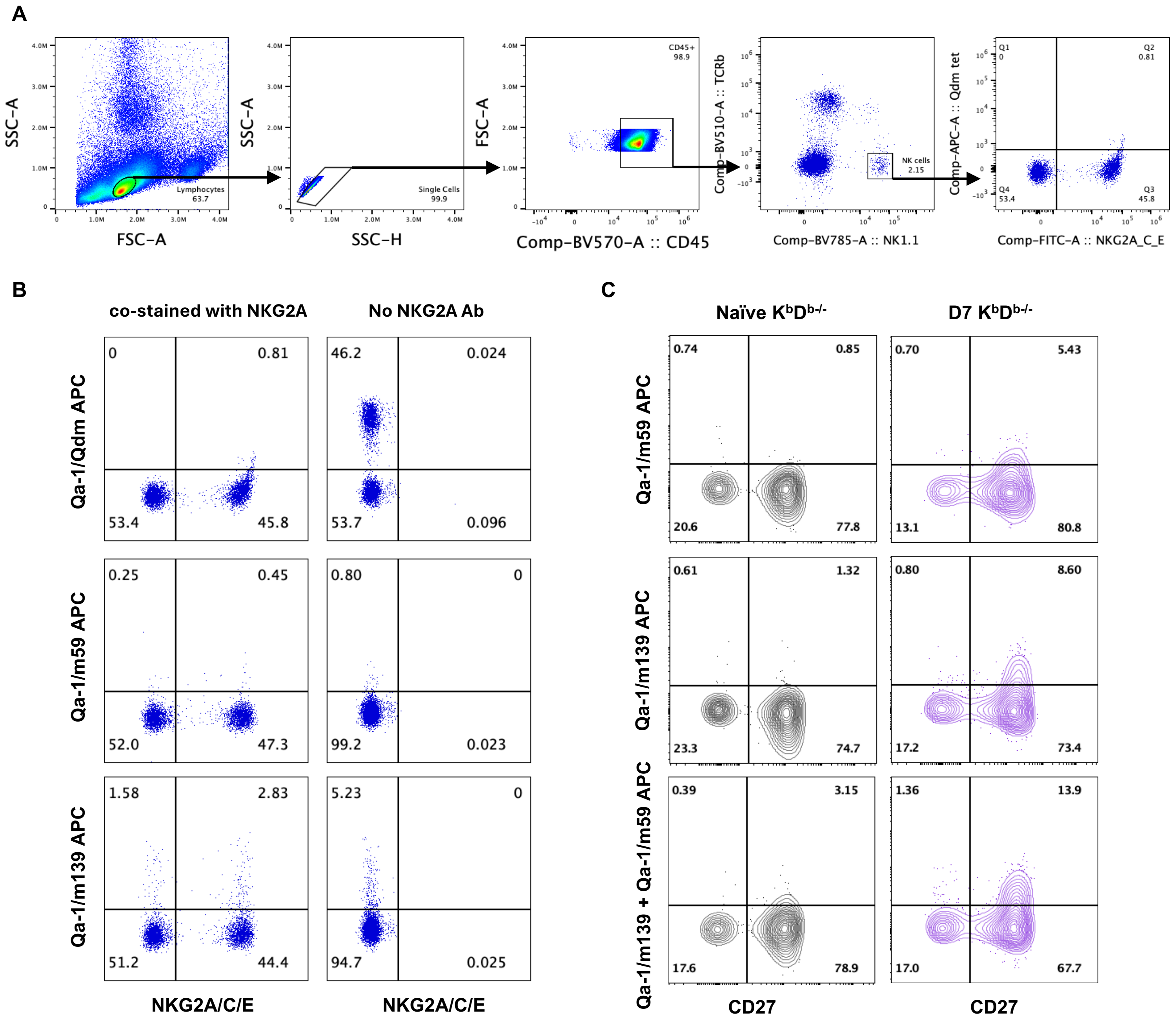

Supplemental Figure 2. (**A**) Gating strategy for NK cells to assess tetramer binding to NKG2A. (**B**) Flow cytometry data for each tetramer stained with and without NKG2A antibody. (**C**) Representative staining of CD27^{+} tetramer^{+} CD8^{+} T cells from the spleens of K^{b}D$^{b-/-}$ mice day 7 post-MCMV infection (5x10^{4} PFU i.p.).

A

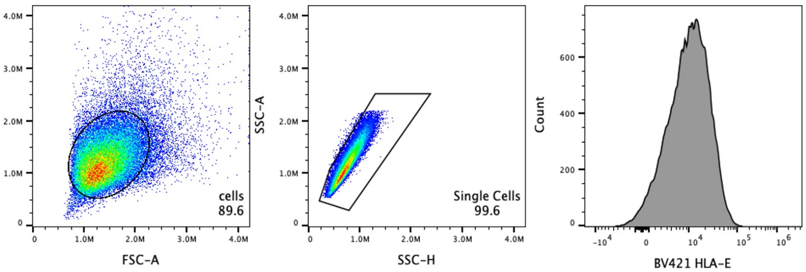

B

K562

K562-HLA-E

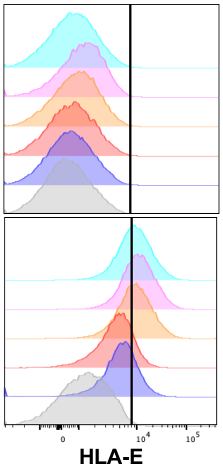

	Sample Name	Subset Name	Count
	LiL K562 Parental.fcs	Single Cells	45159
	LLL K562 Parental.fcs	Single Cells	44664
	VLL K562 Parental.fcs	Single Cells	42091
	DMSO K562 Parental.fcs	Single Cells	19005
	cells K562 Parental.fcs	Single Cells	45425
	unlabeled K562 Parental.fcs	Single Cells	45513

	Sample Name	Subset Name	Count
	LiL K562 HLA-E.fcs	Single Cells	44230
	LLL K562 HLA-E.fcs	Single Cells	43844
	VLL K562 HLA-E.fcs	Single Cells	43895
	DMSO K562 HLA-E.fcs	Single Cells	44305
	cells K562 HLA-E.fcs	Single Cells	25563
	unlabeled.fcs	Single Cells	43254

C

VL9/UL40 (VMAPRTLIL)

LIL: VMAPRTLIL		
Residue		Mean pLDDT Score
P1	VAL	97.764
P2	MET	98.086
P3	ALA	98.012
P4	PRO	95.177
P5	ARG	82.765
P6	THR	96.713
P7	LEU	96.425
P8	ILE	94.678
P9	LEU	96.301
9mer Peptide		94.404

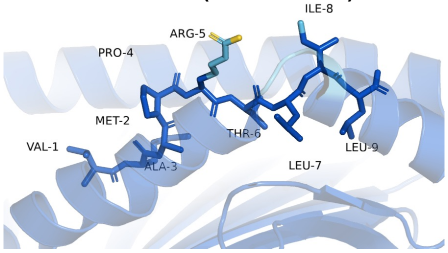

VL9/UL40 (VMAPRTLLL)

LLL: VMAPRTLLL		
Residue		Mean pLDDT Score
P1	VAL	97.504
P2	MET	97.911
P3	ALA	97.772
P4	PRO	94.639
P5	ARG	81.397
P6	THR	96.480
P7	LEU	96.196
P8	LEU	94.654
P9	LEU	96.230
9mer Peptide		94.011

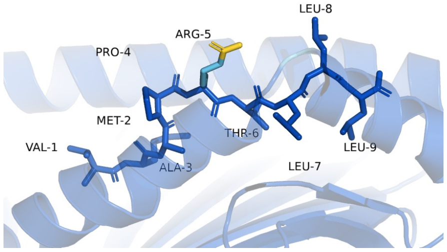

VL9/UL40 (VMAPRTVLL)

VLL: VMAPRTVLL		
Residue		Mean pLDDT Score
P1	VAL	97.494
P2	MET	97.706
P3	ALA	97.598
P4	PRO	94.459
P5	ARG	81.889
P6	THR	95.193
P7	VAL	95.529
P8	LEU	93.623
P9	LEU	96.042
9mer Peptide		93.659

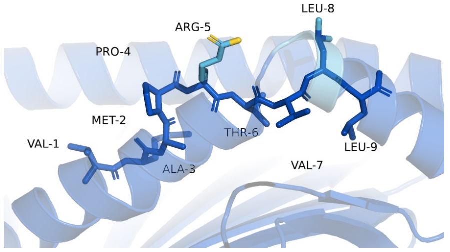

UL75 (QTPPRTTLL)

UL75(Q): QTPPRTTLL		
Residue		Mean pLDDT Score
P1	GLN	78.730
P2	THR	83.793
P3	PRO	82.026
P4	PRO	78.783
P5	ARG	70.087
P6	THR	80.630
P7	THR	85.269
P8	LEU	88.220
P9	LEU	93.743
9mer Peptide		81.979

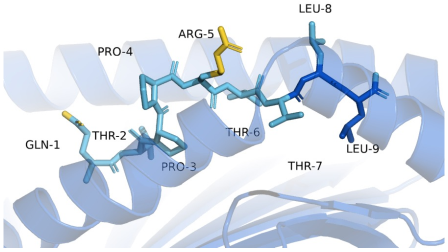

UL75 (TPPRTTLLL)

UL75(T): TPPRTTLLL		
Residue		Mean pLDDT Score
P1	THR	79.937
P2	PRO	80.406
P3	PRO	76.820
P4	ARG	58.325
P5	THR	64.587
P6	THR	68.787
P7	LEU	75.063
P8	LEU	84.164
P9	LEU	90.360
9mer Peptide		74.9632394

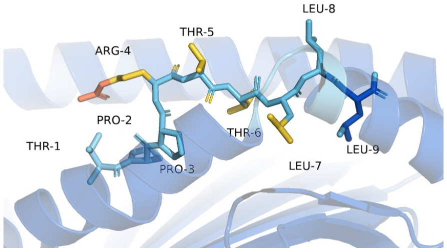

UL147 (TLPVGTELL)

UL147: TLPVGTELL		
Residue		Mean pLDDT Score
P1	THR	90.563
P2	LEU	92.308
P3	PRO	89.204
P4	VAL	75.084
P5	GLY	74.053
P6	THR	69.340
P7	GLU	67.531
P8	LEU	85.910
P9	LEU	92.912
9mer Peptide		82.352727

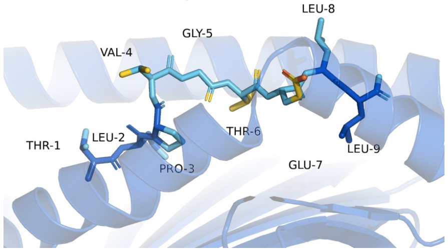

US19 (AAAPFFLGL)

US19: AAAPFFLGL		
Residue		Mean pLDDT Score
P1	ALA	88.900
P2	ALA	87.728
P3	ALA	84.622
P4	PRO	78.764
P5	PHE	68.178
P6	PHE	63.715
P7	LEU	70.348
P8	GLY	87.818
P9	LEU	91.578
9mer Peptide		77.6410769

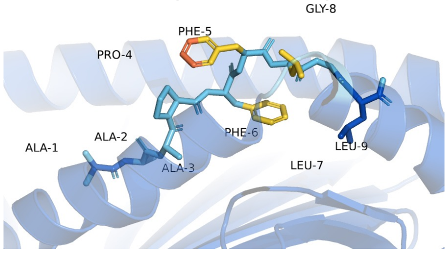

UL97 (FLRLTHPEL)

UL97(F): FLRLTHPEL		
Residue		Mean pLDDT Score
P1	PHE	94.735
P2	LEU	93.784
P3	ARG	82.241
P4	LEU	78.809
P5	THR	74.113
P6	HIS	72.194
P7	PRO	85.801
P8	GLU	85.812
P9	LEU	95.814
9mer Peptide		85.04325

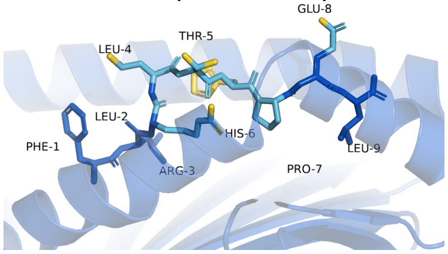

D

Mtb44 (RLPAKAPLL)

Mtb44: RLPAKAPLL		
Residue		Mean pLDDT Score
P1	ARG	88.398
P2	LEU	96.301
P3	PRO	93.984
P4	ALA	86.878
P5	LYS	73.202
P6	ALA	94.800
P7	PRO	96.523
P8	LEU	97.004
P9	LEU	97.567
9mer Peptide		91.271

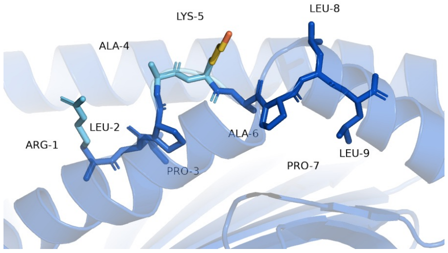

Nsp13 (VMPLSAPTL)

Nsp13: VMPLSAPTL		
Residue		Mean pLDDT Score
P1	VAL	96.356
P2	MET	93.593
P3	PRO	93.916
P4	LEU	83.169
P5	SER	81.738
P6	ALA	93.542
P7	PRO	94.866
P8	THR	94.033
P9	LEU	96.533
9mer Peptide		92.113

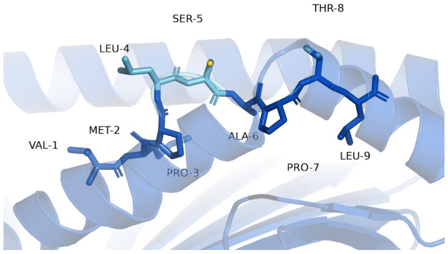

Supplemental Figure 3. (**A**) Gating strategy for HLA-E binding assay performed on K562-HLA-E cell line. Cells were stained with an anti-HLA-E antibody. (**B**) K562 and K562-HLA-E cells were pulsed with peptides, DMSO, or no treatment and HLA-E antibody staining was performed using an anti-HLA-E antibody. (**C**) Alphafold3 was used to model HLA-E-peptide complexes with HCMV peptides that bound to HLA-E in **Fig. 6C** and (**D**) peptides that bind HLA-E during *M. tuberculosis* (Mtb44) or COVID-19 (Nsp13). pLDDT score calculations are presented for each peptide and the peptide residues are colored blue when the score was >90 (very high confidence), cyan when the score was 70-90 (confident prediction), yellow when the score was 50-70 (low confidence, indicating possible disorder or flexibility), or orange when the score was <50 (very low confidence).

Supplemental Table 1. MCMV peptide library was curated by the workflow outlined in **Fig. 1A**. Following motif discovery, peptides that were predicted to exist *in vivo* were organized by Qa-1 binding prediction scores first, then HLA-E binding prediction scores. The MHCI binding predictions were made on 2/10/2024 using the IEDB analysis resource NetMHCpan (ver. 4.1) tool (*34*).

		Qa-1		*HLA-E*	
**MCMV protein**	**Sequence**	**score**	**Percentile Rank**	**score**	**Percentile Rank**
N/A; Qdm	AMAPRTLLL	0.79469	0.01	0.74784	0.01
m09	STSSSPPTF	0.8651	0.01	0.1121	0.17
m10	STDSSLPKF	0.8288	0.01	0.11308	0.17
M32	RTPDPGMSL	0.82835	0.01	0.55773	0.01
m05	STDRSPPTF	0.81586	0.01	0.20716	0.06
m03	KTDDSVWKY	0.81051	0.01	0.02968	1
m07	SSNSSPPLF	0.80816	0.01	0.1084	0.18
m59	RTAGPAPRL	0.80701	0.01	0.24564	0.04
M87	RSPGRQPLF	0.78961	0.01	0.31391	0.01
M25	TVAPSPPKL	0.77754	0.01	0.28264	0.02
m09	TTRPPPTGF	0.77421	0.02	0.06713	0.36
M116	SVSSPSAAF	0.77233	0.02	0.06049	0.41
m29.1	RLFPLTPLL	0.7439	0.03	0.44864	0.01
m14	STPTSPPTL	0.73806	0.03	0.50564	0.01
M25	MTPPRIETL	0.73751	0.03	0.54106	0.01
m10	VSPDRLLQL	0.70388	0.04	0.57497	0.01
m139	RMDDTTYTL	0.71003	0.04	0.53452	0.01
M53	KMAANQPQL	0.60886	0.1	0.30215	0.02
m159	TLPSTTPEL	0.59966	0.12	0.54556	0.01
M72	RLFERGRIL	0.59195	0.13	0.27258	0.02
m59	QLPGYPPRL*	0.56945	0.16	0.43291	0.01
m59	RLLGSAPQL*	0.55914	0.17	0.18143	0.07
M75	SMADLAFLL	0.54061	0.2	0.19429	0.06
m107	RLPARPCYL	0.53068	0.22	0.55289	0.01
m19	ATAPRRPSV	0.52513	0.23	0.07248	0.33
m29	TAAPWSPFL*	0.52563	0.23	0.12641	0.14
m153	RQAQKRPLL	0.50044	0.27	0.3177	0.01
m119.2	AMRAPHPNL	0.47723	0.32	0.22922	0.05
M50	SLRKHGPVL	0.47438	0.32	0.21112	0.06
m29	AAPWSPFLL*	0.454	0.37	0.2833	0.02
N/A; M-SL9	SLQGRTLIL	0.44528	0.39	0.19439	0.06
M70	EMPEKRHVF	0.41227	0.49	0.13019	0.13
m139	QLARRGLSL	0.41156	0.5	0.11553	0.16
N/A; FL9	FYAEATPML	0.36031	0.72	0.14428	0.11
M45	HGIRNASFI	0.07789	6.6	0.01382	2.1

*overlap

Legend:
- **Non Qa-1 binder** (grey)
- **Known Qa-1 binder** (light blue)
- **MCMV predicted Qa-1 binders** (purple)

Supplemental Table 2. Single-cell sample metadata. $CD3^+$ T cells were sorted from the spleens of B6, $K^bD^{b-/-}$, and $K^bD^{b-/-}Qa\text{-}1^{-/-}$ mice at naïve state or following long-term MCMV infection and subjected to scRNA/Totalseq and scTCRseq. Data is pooled from 2 independent experiments, *n* = 2-3 mice per group.

Sample	Count
B6 naïve	451
$K^bD^{b-/-}$ naïve	1467
$K^bD^{b-/-}Qa\text{-}1^{-/-}$ naïve	2350
B6 infected 1	2005
$K^bD^{b-/-}$ infected 1	1790
$K^bD^{b-/-}Qa\text{-}1^{-/-}$ infected 1	1594

Supplemental Table 3. Hashtag and Cite-seq antibodies used for single-cell experiments.

id	name	read	pattern	sequence	feature_type
HT1	HT1	R2	5PNNNNNNNNNN(BC)NNNNNNNNN	ACCCACCAGTAAGAC	Antibody Capture
HT2	HT2	R2	5PNNNNNNNNNN(BC)NNNNNNNNN	GGTCGAGAGCATTCA	Antibody Capture
HT3	HT3	R2	5PNNNNNNNNNN(BC)NNNNNNNNN	CTTGCCGCATGTCAT	Antibody Capture
HT4	HT4	R2	5PNNNNNNNNNN(BC)NNNNNNNNN	AAAGCATTCTTCACG	Antibody Capture
HT5	HT5	R2	5PNNNNNNNNNN(BC)NNNNNNNNN	CTTTGTCTTTGTGAG	Antibody Capture
HT6	HT6	R2	5PNNNNNNNNNN(BC)NNNNNNNNN	TATGCTGCCACGGTA	Antibody Capture
HT7	HT7	R2	5PNNNNNNNNNN(BC)NNNNNNNNN	GAGTCTGCCAGTATC	Antibody Capture
HT8	HT8	R2	5PNNNNNNNNNN(BC)NNNNNNNNN	TATAGAACGCCAGGC	Antibody Capture
HT9	HT9	R2	5PNNNNNNNNNN(BC)NNNNNNNNN	TGCCTATGAAACAAG	Antibody Capture
HT10	HT10	R2	5PNNNNNNNNNN(BC)NNNNNNNNN	CCGATTGTAACAGAC	Antibody Capture
HT11	HT11	R2	5PNNNNNNNNNN(BC)NNNNNNNNN	GCTTACCGAATTAAC	Antibody Capture
HT12	HT12	R2	5PNNNNNNNNNN(BC)NNNNNNNNN	CTGCAAATATAACGG	Antibody Capture
HT13	HT13	R2	5PNNNNNNNNNN(BC)NNNNNNNNN	CTACATTGCGATTTG	Antibody Capture
HT14	HT14	R2	5PNNNNNNNNNN(BC)NNNNNNNNN	CTTTCGCCAACTCTG	Antibody Capture
HT15	HT15	R2	5PNNNNNNNNNN(BC)NNNNNNNNN	CCCTCTCTGGATTCT	Antibody Capture
HT16	HT16	R2	5PNNNNNNNNNN(BC)NNNNNNNNN	GATGTATCCGTTGTC	Antibody Capture
TCRVG1.1	TCRVG1.1	R2	5PNNNNNNNNNN(BC)NNNNNNNNN	TCGTTTAACCAGCCT	Antibody Capture
CD28	CD28	R2	5PNNNNNNNNNN(BC)NNNNNNNNN	ATTAAGAGCGTGTTG	Antibody Capture
CD38	CD38	R2	5PNNNNNNNNNN(BC)NNNNNNNNN	CGTATCCGTCTCCTA	Antibody Capture
CD16_CD32	CD16_CD32	R2	5PNNNNNNNNNN(BC)NNNNNNNNN	TTCGATGCTGGAGCA	Antibody Capture
CD45.2	CD45.2	R2	5PNNNNNNNNNN(BC)NNNNNNNNN	CACCGTCATTCAACC	Antibody Capture
CD31	CD31	R2	5PNNNNNNNNNN(BC)NNNNNNNNN	GCTGTAGTATCATGT	Antibody Capture
TCRVG3	TCRVG3	R2	5PNNNNNNNNNN(BC)NNNNNNNNN	TCGTGGTCCCTTTCT	Antibody Capture
BST2.PDCA1.CD317	BST2.PDCA1.CD317	R2	5PNNNNNNNNNN(BC)NNNNNNNNN	TGTGGTAGCCCTTGT	Antibody Capture
FCERA	FCERA	R2	5PNNNNNNNNNN(BC)NNNNNNNNN	AGTCACCTCGAAGCT	Antibody Capture
CD103	CD103	R2	5PNNNNNNNNNN(BC)NNNNNNNNN	TTCATTAGCCCGCTG	Antibody Capture
NEUROPILIN1.CD304	NEUROPILIN1.CD304	R2	5PNNNNNNNNNN(BC)NNNNNNNNN	CCAGCTCATTCAACG	Antibody Capture
CD3E	CD3E	R2	5PNNNNNNNNNN(BC)NNNNNNNNN	TAATGCCAGTTGTGC	Antibody Capture
CD80	CD80	R2	5PNNNNNNNNNN(BC)NNNNNNNNN	GACCCGGTGTCATTT	Antibody Capture
CD3	CD3	R2	5PNNNNNNNNNN(BC)NNNNNNNNN	GTATGTCCGCTCGAT	Antibody Capture
CD137	CD137	R2	5PNNNNNNNNNN(BC)NNNNNNNNN	TCCCTGTATAGATGA	Antibody Capture
CD94	CD94	R2	5PNNNNNNNNNN(BC)NNNNNNNNN	CACAGTTGTCCGTGT	Antibody Capture
LY6G	LY6G	R2	5PNNNNNNNNNN(BC)NNNNNNNNN	ACATTGACGCAACTA	Antibody Capture
CD1D	CD1D	R2	5PNNNNNNNNNN(BC)NNNNNNNNN	CAACTTGGCCGAATC	Antibody Capture
P2X7R	P2X7R	R2	5PNNNNNNNNNN(BC)NNNNNNNNN	TGCTTCATTCATGTG	Antibody Capture
TIGIT	TIGIT	R2	5PNNNNNNNNNN(BC)NNNNNNNNN	GAAAGTCGCCAACAG	Antibody Capture
CD138	CD138	R2	5PNNNNNNNNNN(BC)NNNNNNNNN	GCGTTTGTATGTACT	Antibody Capture
CCR6	CCR6	R2	5PNNNNNNNNNN(BC)NNNNNNNNN	CTCTCTGCATTCCTC	Antibody Capture
NKP46	NKP46	R2	5PNNNNNNNNNN(BC)NNNNNNNNN	CCCTTTCACCTCGAA	Antibody Capture
KIT	KIT	R2	5PNNNNNNNNNN(BC)NNNNNNNNN	TGCATGTCATCGGTG	Antibody Capture
CD61	CD61	R2	5PNNNNNNNNNN(BC)NNNNNNNNN	TTCTTTACCCGCCTG	Antibody Capture
KLRG1	KLRG1	R2	5PNNNNNNNNNN(BC)NNNNNNNNN	GTAGTAGGCTAGACC	Antibody Capture
CCR4.CD194	CCR4.CD194	R2	5PNNNNNNNNNN(BC)NNNNNNNNN	TTCATGTGTTTGTGC	Antibody Capture
CD45	CD45	R2	5PNNNNNNNNNN(BC)NNNNNNNNN	TGGCTATGGAGCAGA	Antibody Capture
THY1.2	THY1.2	R2	5PNNNNNNNNNN(BC)NNNNNNNNN	CCGATCAGCCGTTTA	Antibody Capture
LY108	LY108	R2	5PNNNNNNNNNN(BC)NNNNNNNNN	CGATTCTTTGCGAGT	Antibody Capture
IL21R	IL21R	R2	5PNNNNNNNNNN(BC)NNNNNNNNN	GATTCCGACAGTAGA	Antibody Capture
CCR7	CCR7	R2	5PNNNNNNNNNN(BC)NNNNNNNNN	TTATTAACAGCCCAC	Antibody Capture
TNFRSF25.DR3	TNFRSF25.DR3	R2	5PNNNNNNNNNN(BC)NNNNNNNNN	GCTTGGGCAATTAAG	Antibody Capture
CD8A	CD8A	R2	5PNNNNNNNNNN(BC)NNNNNNNNN	TACCCGTAATAGCGT	Antibody Capture
CD5	CD5	R2	5PNNNNNNNNNN(BC)NNNNNNNNN	CAGCTCAGTGTGTTG	Antibody Capture
IL2RB.CD122	IL2RB.CD122	R2	5PNNNNNNNNNN(BC)NNNNNNNNN	GGTATGCGACACTTA	Antibody Capture
BTLA.CD272	BTLA.CD272	R2	5PNNNNNNNNNN(BC)NNNNNNNNN	TGACCCTATTGAGAA	Antibody Capture
CD19	CD19	R2	5PNNNNNNNNNN(BC)NNNNNNNNN	ATCAGCCATGTCAGT	Antibody Capture
CD160	CD160	R2	5PNNNNNNNNNN(BC)NNNNNNNNN	GCGTATGTCAGTACC	Antibody Capture
CD45.1	CD45.1	R2	5PNNNNNNNNNN(BC)NNNNNNNNN	CCTATGGACTTGGAC	Antibody Capture
IL7RA.CD127	IL7RA.CD127	R2	5PNNNNNNNNNN(BC)NNNNNNNNN	GTGTGAGGCACTCTT	Antibody Capture
TCRVA2	TCRVA2	R2	5PNNNNNNNNNN(BC)NNNNNNNNN	TTGACCCGCTACGAA	Antibody Capture
TCRVA8.3	TCRVA8.3	R2	5PNNNNNNNNNN(BC)NNNNNNNNN	TTAGAGTCGCTTGGA	Antibody Capture
F480	F480	R2	5PNNNNNNNNNN(BC)NNNNNNNNN	TTAACTTCAGCCCGT	Antibody Capture
CD45RB	CD45RB	R2	5PNNNNNNNNNN(BC)NNNNNNNNN	TTGTATCTCCCTTGG	Antibody Capture

ICOS.CD278	ICOS.CD278	R2	5PNNNNNNNNNN(BC)NNNNNNNNN	CGCGCACCCATTAAA	Antibody Capture
LAG3.CD223	LAG3.CD223	R2	5PNNNNNNNNNN(BC)NNNNNNNNN	ATTCCGTCCCTAAGG	Antibody Capture
CXCR3	CXCR3	R2	5PNNNNNNNNNN(BC)NNNNNNNNN	GTTCACGCCGTGTTA	Antibody Capture
SCA1	SCA1	R2	5PNNNNNNNNNN(BC)NNNNNNNNN	TTCCTTTCCTACGCA	Antibody Capture
IL1RL1.ST2	IL1RL1.ST2	R2	5PNNNNNNNNNN(BC)NNNNNNNNN	GCGATGGAGCATGTT	Antibody Capture
GITR.CD357	GITR.CD357	R2	5PNNNNNNNNNN(BC)NNNNNNNNN	GGCACTCTGTAACAT	Antibody Capture
CD39	CD39	R2	5PNNNNNNNNNN(BC)NNNNNNNNN	GCGTATTTAACCCGT	Antibody Capture
CD81	CD81	R2	5PNNNNNNNNNN(BC)NNNNNNNNN	TTGTCACCAACTTCC	Antibody Capture
ITB7	ITB7	R2	5PNNNNNNNNNN(BC)NNNNNNNNN	TCCTTGGATGTACCG	Antibody Capture
Isotype.Rat.IgG1bl	Isotype.Rat.IgG1bl	R2	5PNNNNNNNNNN(BC)NNNNNNNNN	GGGAGCGATTCAACT	Antibody Capture
CD86	CD86	R2	5PNNNNNNNNNN(BC)NNNNNNNNN	CTGGATTTGTGTATC	Antibody Capture
TCRGD	TCRGD	R2	5PNNNNNNNNNN(BC)NNNNNNNNN	AACCCAAATAGCTGA	Antibody Capture
ITA6.CD49F	ITA6.CD49F	R2	5PNNNNNNNNNN(BC)NNNNNNNNN	TTCCGAGGATGATCT	Antibody Capture
CD69	CD69	R2	5PNNNNNNNNNN(BC)NNNNNNNNN	TTGTATTCCGCCATT	Antibody Capture
TCRB	TCRB	R2	5PNNNNNNNNNN(BC)NNNNNNNNN	TCCTATGGGACTCAG	Antibody Capture
LY6C1_LY6C2	LY6C1_LY6C2	R2	5PNNNNNNNNNN(BC)NNNNNNNNN	AAGTCGTGAGGCATG	Antibody Capture
CD48	CD48	R2	5PNNNNNNNNNN(BC)NNNNNNNNN	AGAACCGCCGTAGTT	Antibody Capture
HVEM.CD270	HVEM.CD270	R2	5PNNNNNNNNNN(BC)NNNNNNNNN	GATCCGTGTTGCCTA	Antibody Capture
CD49A	CD49A	R2	5PNNNNNNNNNN(BC)NNNNNNNNN	CCATTCATTTGTGGC	Antibody Capture
CD49B	CD49B	R2	5PNNNNNNNNNN(BC)NNNNNNNNN	CGCGTTAGTAGAGTC	Antibody Capture
CD29	CD29	R2	5PNNNNNNNNNN(BC)NNNNNNNNN	ACGCATTCCTTGTGT	Antibody Capture
Isotype.Hamster.IgG	Isotype.Hamster.IgG	R2	5PNNNNNNNNNN(BC)NNNNNNNNN	CCTGTCATTAAGACT	Antibody Capture
IL4RA.CD124	IL4RA.CD124	R2	5PNNNNNNNNNN(BC)NNNNNNNNN	GAACCGTAGTATAAC	Antibody Capture
CD44	CD44	R2	5PNNNNNNNNNN(BC)NNNNNNNNN	TGGCTTCAGGTCCTA	Antibody Capture
TCRVB8.1_TCRVB8.2	TCRVB8.1_TCRVB8.2	R2	5PNNNNNNNNNN(BC)NNNNNNNNN	ACTATCCGTTGTGCT	Antibody Capture
TCRVA8.3bis	TCRVA8.3bis	R2	5PNNNNNNNNNN(BC)NNNNNNNNN	CTTTCCCTAGTATGC	Antibody Capture
CXCR5	CXCR5	R2	5PNNNNNNNNNN(BC)NNNNNNNNN	ACGTAGTCACCTAGT	Antibody Capture
CD27	CD27	R2	5PNNNNNNNNNN(BC)NNNNNNNNN	CAAGGTATGTCACTG	Antibody Capture
CD24	CD24	R2	5PNNNNNNNNNN(BC)NNNNNNNNN	TATATCTTTGCCGCA	Antibody Capture
ITAM.CD11B	ITAM.CD11B	R2	5PNNNNNNNNNN(BC)NNNNNNNNN	TGAAGGCTCATTTGT	Antibody Capture
CD11A	CD11A	R2	5PNNNNNNNNNN(BC)NNNNNNNNN	AGAGTCTCCCTTTAG	Antibody Capture
I.A_I.E	I.A_I.E	R2	5PNNNNNNNNNN(BC)NNNNNNNNN	GGTCACCAGTATGAT	Antibody Capture
CD30	CD30	R2	5PNNNNNNNNNN(BC)NNNNNNNNN	ATCATTCCCGCTTAA	Antibody Capture
CD62L	CD62L	R2	5PNNNNNNNNNN(BC)NNNNNNNNN	TGGGCCTAAGTCATC	Antibody Capture
FASL.CD178	FASL.CD178	R2	5PNNNNNNNNNN(BC)NNNNNNNNN	GTCACGTAGTATCTT	Antibody Capture
CD83	CD83	R2	5PNNNNNNNNNN(BC)NNNNNNNNN	TCTCAGGCTTCCTAG	Antibody Capture
PD1L1.CD274	PD1L1.CD274	R2	5PNNNNNNNNNN(BC)NNNNNNNNN	TCGATTCCACCAACT	Antibody Capture
VISTA	VISTA	R2	5PNNNNNNNNNN(BC)NNNNNNNNN	ACATTTCCCTTGCCT	Antibody Capture
Isotype.Mouse.IgG2ak	Isotype.Mouse.IgG2ak	R2	5PNNNNNNNNNN(BC)NNNNNNNNN	CTCCTACCTAAACTG	Antibody Capture
Isotype.Mouse.IgG1k	Isotype.Mouse.IgG1k	R2	5PNNNNNNNNNN(BC)NNNNNNNNN	GCCGGACGACATTAA	Antibody Capture
Isotype.Mouse.IgG2bk	Isotype.Mouse.IgG2bk	R2	5PNNNNNNNNNN(BC)NNNNNNNNN	ATATGTATCACGCGA	Antibody Capture
TCRVB5.1_TCRVB5.2	TCRVB5.1_TCRVB5.2	R2	5PNNNNNNNNNN(BC)NNNNNNNNN	CTCAACAGTATTCTG	Antibody Capture
CD9	CD9	R2	5PNNNNNNNNNN(BC)NNNNNNNNN	TAGCAGTCACTCCTA	Antibody Capture
ITAX.CD11C	ITAX.CD11C	R2	5PNNNNNNNNNN(BC)NNNNNNNNN	GTTATGGACGCTTGC	Antibody Capture
CD200R	CD200R	R2	5PNNNNNNNNNN(BC)NNNNNNNNN	ATTCTTTCCCTCTGT	Antibody Capture
THY1.1.CD90.1	THY1.1.CD90.1	R2	5PNNNNNNNNNN(BC)NNNNNNNNN	AGTATGGGATGCAAT	Antibody Capture
OX40.CD134	OX40.CD134	R2	5PNNNNNNNNNN(BC)NNNNNNNNN	CTCACCTACCTATGG	Antibody Capture
CD200	CD200	R2	5PNNNNNNNNNN(BC)NNNNNNNNN	TCAATTCCGGTAGTC	Antibody Capture
IL2RA.CD25	IL2RA.CD25	R2	5PNNNNNNNNNN(BC)NNNNNNNNN	ACCATGAGACACAGT	Antibody Capture
KLRBC_NK1.1	KLRBC_NK1.1	R2	5PNNNNNNNNNN(BC)NNNNNNNNN	GTAACATTACTCGTC	Antibody Capture
ITA4.CD49D	ITA4.CD49D	R2	5PNNNNNNNNNN(BC)NNNNNNNNN	CGCTTGGACGCTTAA	Antibody Capture
B220	B220	R2	5PNNNNNNNNNN(BC)NNNNNNNNN	CCTACACCTCATAAT	Antibody Capture
GR1_LY6G_LY6C1_LY6C2	GR1_LY6G_LY6C1_LY6C2	R2	5PNNNNNNNNNN(BC)NNNNNNNNN	TAGTGTATGGACACG	Antibody Capture
CD55.DAF	CD55.DAF	R2	5PNNNNNNNNNN(BC)NNNNNNNNN	ATTGTTGTCAGACCA	Antibody Capture
CD2	CD2	R2	5PNNNNNNNNNN(BC)NNNNNNNNN	TTGCCGTGTGTTTAA	Antibody Capture
CD4	CD4	R2	5PNNNNNNNNNN(BC)NNNNNNNNN	AACAAGACCCTTGAG	Antibody Capture
PDCD1.PD1.CD279	PDCD1.PD1.CD279	R2	5PNNNNNNNNNN(BC)NNNNNNNNN	GAAAGTCAAAGCACT	Antibody Capture
HAVCR2	HAVCR2	R2	5PNNNNNNNNNN(BC)NNNNNNNNN	ATTGGCACTCAGATG	Antibody Capture
Isotype.Rat.IgG1bk	Isotype.Rat.IgG1bk	R2	5PNNNNNNNNNN(BC)NNNNNNNNN	ATCAGATGCCCTCAT	Antibody Capture
Isotype.Rat.IgG2ak	Isotype.Rat.IgG2ak	R2	5PNNNNNNNNNN(BC)NNNNNNNNN	AAGTCAGGTTCGTTT	Antibody Capture
Isotype.Rat.IgG2ck	Isotype.Rat.IgG2ck	R2	5PNNNNNNNNNN(BC)NNNNNNNNN	TCCAGGCTAGTCATT	Antibody Capture
Isotype.Rat.IgG2bk	Isotype.Rat.IgG2bk	R2	5PNNNNNNNNNN(BC)NNNNNNNNN	GATTCTTGACGACCT	Antibody Capture

CD43	CD43	R2	5PNNNNNNNNNN(BC)NNNNNNNNN	TTGGAGGGTTGTGCT	Antibody Capture
CX3CR1	CX3CR1	R2	5PNNNNNNNNNN(BC)NNNNNNNNN	CACTCTCAGTCCTAT	Antibody Capture
CXCR2	CXCR2	R2	5PNNNNNNNNNN(BC)NNNNNNNNN	TTTCCTTGTAGAGCG	Antibody Capture
CXCR6.CD186	CXCR6.CD186	R2	5PNNNNNNNNNN(BC)NNNNNNNNN	TGTCAGGTTGTATTC	Antibody Capture
CCR2.CD192	CCR2.CD192	R2	5PNNNNNNNNNN(BC)NNNNNNNNN	AGTGCGATCTGCAAC	Antibody Capture
CCR8	CCR8	R2	5PNNNNNNNNNN(BC)NNNNNNNNN	ATCTCCGTTGTGCGA	Antibody Capture
CD20	CD20	R2	5PNNNNNNNNNN(BC)NNNNNNNNN	TCCACTCCCTGTATA	Antibody Capture
FAS.CD95	FAS.CD95	R2	5PNNNNNNNNNN(BC)NNNNNNNNN	CACATCGTTTGTGTA	Antibody Capture
CD34	CD34	R2	5PNNNNNNNNNN(BC)NNNNNNNNN	AAACTCAGGTCCTTC	Antibody Capture
SLAM.CD150	SLAM.CD150	R2	5PNNNNNNNNNN(BC)NNNNNNNNN	CAACGCCTAGAAACC	Antibody Capture
TER119	TER119	R2	5PNNNNNNNNNN(BC)NNNNNNNNN	GCGCGTTTGTGCTAT	Antibody Capture
FR4	FR4	R2	5PNNNNNNNNNN(BC)NNNNNNNNN	TGATCTAAGGTATGC	Antibody Capture
CD120B	CD120B	R2	5PNNNNNNNNNN(BC)NNNNNNNNN	GAAGCTGTATCCGAA	Antibody Capture
CD226.DNAM1	CD226.DNAM1	R2	5PNNNNNNNNNN(BC)NNNNNNNNN	CGGTATCCGTCAGTT	Antibody Capture
CD155.PVR	CD155.PVR	R2	5PNNNNNNNNNN(BC)NNNNNNNNN	TAGCTTGGGATTAAG	Antibody Capture
CD73.5NTD	CD73.5NTD	R2	5PNNNNNNNNNN(BC)NNNNNNNNN	ACACTTAACGTCTGG	Antibody Capture
PD1L2.CD273	PD1L2.CD273	R2	5PNNNNNNNNNN(BC)NNNNNNNNN	CACTCCTTGTAGTCA	Antibody Capture
TCRVG2	TCRVG2	R2	5PNNNNNNNNNN(BC)NNNNNNNNN	AAGCTGCACCGTAAT	Antibody Capture
LY49A	LY49A	R2	5PNNNNNNNNNN(BC)NNNNNNNNN	AATTCCGTCAGATGA	Antibody Capture
ICAM1	ICAM1	R2	5PNNNNNNNNNN(BC)NNNNNNNNN	ATAACCGACACAGTG	Antibody Capture
CD8B	CD8B	R2	5PNNNNNNNNNN(BC)NNNNNNNNN	TTCCCTCTATGGAGC	Antibody Capture

Supplemental Table 4. HCMV peptide library was based on a list of CMV peptides from a previous study (*45*). Following motif discovery with MCMV peptides that bind Qa-1 (**Fig. 1C**), peptides were organized by HLA-E binding prediction scores first, then Qa-1 binding prediction scores. The MHC binding predictions were made on 2/10/2024 using the IEDB analysis resource NetMHCpan (ver. 4.1) tool (*34*).

		HLA-E		*Qa-1*	
**HCMV Protein**	**Sequence**	**ic50**	**Percentile rank**	**ic50**	**Percentile rank**
UL40	VMAPRTLIL	87.81	0.01	104.99	0.02
UL88	QSATRGDSD	494.8	0.01	319.77	0.1
UL120	VLPHRTQFL	52.53	0.01	47.39	0.02
UL102	TGAARSFFF	88.61	0.01	77.26	0.02
UL40	VMAPRTLLL	3954.31	0.02	309.36	0.09
UL75	QTPPRTTLL*	4307.78	0.02	1695.83	0.93
UL75	TPPRTTLLL*	6623.82	0.04	765.36	0.33
UL87	SCAPRTSRL	8507.56	0.07	163.94	0.04
UL87	ACLPRDLSL	9041.52	0.07	158.13	0.04
UL29/UL28	ATAGRWLPL	11528.45	0.13	457.82	0.17
UL84	ATFPRDALL	13129.91	0.2	271.18	0.08
IE2 (UL122)	PGASATPEL	13887.12	0.22	1235.58	0.62
UL40	VMAPRTVLL	14816.47	0.25	6977.22	5.2
UL147	TLPVGTELL	16005.77	0.31	4273.85	2.8
UL57	KFAALQEQG	17212.7	0.38	1121.89	0.55
UL97	FLRLTHPEL	17560.33	0.4	2090.39	1.3
UL78	ELATRTLLT	17637.44	0.42	1550.12	0.84
UL150	ALHRRPDSL	21279.1	0.72	1459.89	0.77
US19	AAAPFFLGL	23976.61	1.2	3057.47	1.9
US23	RPGSTMPEL	24685.51	1.3	4585.39	3.1
IRS1	VMTERQSQL	25660.45	1.5	3351.53	2.2
US26	RAADSLPQL	28287.68	2.1	7544.37	5.7
UL114	AWARRGVLL	29850.81	2.6	10244.17	8.5
UL71	QLAQRLCEL	33032.62	4.2	12354.64	12
UL71	RYKESAPQE	33610.91	4.6	643.94	0.27
UL79	KMSRGQPLS	34284.52	5	16144.56	17
UL98	RLLDLAPNY	35876.13	6.4	16695.8	18
UL97	LLDRRGLDE	37011.27	7.6	17047.56	19
UL50	QATRRILKL	39265.74	12	12512.17	13
RL12	TISSTTNTL	40063.09	14	8523.31	6.7
UL77	GPSPRTLLQ	41063.7	16	7322.64	5.5
UL32	SPPSQTPEQ	40840.38	16	6725.21	4.9
UL88	QLIRRVLPP	40712.44	16	11540.18	10
UL26	QLLERGLLH	41313.26	17	14355.53	15
UL37	NLERRGAQL	42520.04	21	20446.27	24
US28	TPTTTTAEL	42395.54	21	21518.51	26
UL76	GNARRGLAR	42577.58	21	11532.19	10
UL48	ELLGKATQQ	42490.13	21	11431.31	9.9
UL124	TLPSTVNST	43620.23	25	24416.95	31
UL93	ARCPRTGLW	44096.16	28	32286.7	50
UL72	TLPQSTEEK	44567.67	30	23813.22	30
IRS1	AEAARRLLP	44945.38	33	26623.46	36
UL37	QMALIQPAS	46131.79	43	26961.46	37
US24	FLNDTTPEG	46161.25	43	24421.44	31
US28	ASVPCTLLT	46309.83	44	33607.63	55
US24	ELARYGVSR	46379.02	45	29738.3	43
US3	KYAACVPQV	47287.54	58	33895.76	55
UL86	QLAEEPLSA	47563.11	63	31313.05	47
UL49	QQARGGLAA	48402.05	83	36020.82	63

*overlap

Known HLA-E binders

HCMV peptides

Supplemental File 1. Tabulated data file with all data points from mouse experiments.

Supplemental File 2. Output data from FIMO, IEDB-AR, and PROSPER analyses.

Supplemental Files 3-8. Bulk TCR alpha seq data.

Supplemental Files 9-15. Bulk TCR beta seq data.
